# Ribosomal Protein S6: A Potential Therapeutic Target against Cancer?

**DOI:** 10.3390/ijms23010048

**Published:** 2021-12-21

**Authors:** Yong Weon Yi, Kyu Sic You, Jeong-Soo Park, Seok-Geun Lee, Yeon-Sun Seong

**Affiliations:** 1 Department of Biochemistry, College of Medicine, Dankook University, Cheonan 31116, Chungcheongnam-do, Korea; yongweon_yi@dankook.ac.kr (Y.W.Y.); 12200706@dankook.ac.kr (K.S.Y.); jeongsp@dankook.ac.kr (J.-S.P.); 2 Department of Nanobiomedical Science, Dankook University, Cheonan 31116, Chungcheongnam-do, Korea; 3 Graduate School of Convergence Medical Science, Dankook University, Cheonan 31116, Chungcheongnam-do, Korea; 4 Graduate School, Kyung Hee University, Seoul 02447, Korea

**Keywords:** ribosomal protein S6, biomarker, anticancer, cancer therapeutics, drug resistance, therapeutic target

## Abstract

Ribosomal protein S6 (RPS6) is a component of the 40S small ribosomal subunit and participates in the control of mRNA translation. Additionally, phospho (p)-RPS6 has been recognized as a surrogate marker for the activated PI3K/AKT/mTORC1 pathway, which occurs in many cancer types. However, downstream mechanisms regulated by RPS6 or p-RPS remains elusive, and the therapeutic implication of RPS6 is underappreciated despite an approximately half a century history of research on this protein. In addition, substantial evidence from RPS6 knockdown experiments suggests the potential role of RPS6 in maintaining cancer cell proliferation. This motivates us to investigate the current knowledge of RPS6 functions in cancer. In this review article, we reviewed the current information about the transcriptional regulation, upstream regulators, and extra-ribosomal roles of RPS6, with a focus on its involvement in cancer. We also discussed the therapeutic potential of RPS6 in cancer.

## 1. Background

Eukaryotic ribosome (80S) consists of the 60S large and 40S small ribosomal subunits, which contain four ribosomal RNAs (rRNAs; 5S, 5.8S, and 28S rRNAs in the large subunit and 18S rRNA in the small subunit) and 79 ribosomal proteins (RPs) [1,2]. RPs are RNA-binding proteins and are primarily involved in the regulation of protein translation [3]. RPs also function in ribosome biogenesis in the nucleolus. They act as RNA chaperones to ensure the stabilization and correct folding of rRNAs to assemble ribosomal subunits. Dysregulation of ribosome biogenesis may result in tumorigenesis [4]. RPs also have multiple extra-ribosomal functions, including regulation of cell-cycle arrest, cell proliferation, cell migration and invasion, apoptosis, DNA damage repair, malignant transformation, and tumorigenesis via both p53/mouse double-minute 2 homolog (MDM2)-dependent and p53-independent mechanisms [3]. For example, ribosomal protein L5 (RPL5) [5], RPL11 [6,7], and RPL23 [8,9] exert anticancer effects through the activation of the tumor suppressor p53 by specifically binding to its inhibitor MDM2.

Ribosomal protein S6 (RPS6), also known as phosphoprotein NP33 [10], is a component of the 40S small ribosomal subunit as a ribosomal RNA-binding protein [11,12]. RPS6 is evolutionarily conserved across eukaryotes from yeast to vertebrates [12] and plays important roles in ribosome biogenesis, protein translation, cell proliferation (increase in cell number), cell growth (increase in cell mass/volume), DNA repair, apoptosis, cell differentiation, and glucose metabolism in both normal and cancer cells [13,14,15]. RPS6 is the first ribosomal protein identified to be phosphorylated by protein kinases [16] and is one of the only two RPs known to be phosphorylated [17].

RPS6 is used as a readout of the mechanistic/mammalian target of rapamycin complex 1 (mTORC1) activity in many diseases, including cancers, since alleviated activity of mTORC1, an upstream regulator of RPS6, is commonly found in many types of cancer cells [2,18,19,20]. However, considering the almost five-decade-long history of research on RPS6, the functions and therapeutic implications of this protein and its downstream regulatory pathways have not been well appreciated yet. Recently, we found that dual inhibition of epidermal growth factor receptor (EGFR) and MET kinase activities resulted in the synergistic anti-proliferation effect through downregulation of RPS6 in triple-negative breast cancer (TNBC) cells [21]. In addition, knockdown of RPS6 was enough to suppress the proliferation of TNBC cells in vitro. These results motivate us to investigate previous studies on the therapeutic potential of RPS6 targeting. In this review article, we analyzed the roles of RPS6 in tumorigenesis and evaluated the potential of using it as an anticancer therapeutic target.

## 2. Regulation of RPS6

### 2.1. Transcriptional Regulation of RPS6

The human *RPS6* gene is located on chromosome 9p21, comprises six exons [22], and encodes a protein of 249 amino acids [23,24]. Contrary to rRNAs, which are transcribed by RNA polymerase I or III, RP genes are transcribed by RNA polymerase II [1]. Following transcription, the produced RP mRNAs are transported from the nucleoplasm to the cytoplasm for translation, after which the de novo formed RPs are translocated into the nucleus and concentrated in the nucleolus. Interestingly, the promoter regions of most RP genes are phylogenetically well conserved [1].

The promoters of the human *RPS6* and mouse *Rps6* genes share 80% sequence similarity [25], lack a consensus TATA box, and have high GpC content [22,25]. Consensus binding sites for specificity protein 1 (SP1) have been found in the upstream regions and within the first introns of both human *RPS6* and mouse *Rps6* genes [22,25]. The SP1 sites in the human *RPS6* gene have been reported to minimally contribute to the transcription of the gene in COS-1 cells [26]. Additionally, the human *RPS6* promoter contains four putative binding sites for GA-binding protein (GABP), a member of the ETS DNA-binding protein family. Potential binding sequences for CCAAT/enhancer binding protein (C/EBP) and the DNA-binding protein Ikaros have also been identified. In addition, the recombinant Yin-Yang 1 (YY1) protein has been shown to bind to the consensus binding site in the first intron of the human *RPS6* gene. In fact, upstream GABP and SP1 sites and downstream YY1 sites are common features of several RP gene promoters [27]. However, the functional implications of GABP and YY1 have not been determined yet [26,28,29]. 

Chromatin immunoprecipitation experiments have shown that the MYC protein binds to the E-box motif in the *TSC2* and *RPS6* genes [30]. Interestingly, MYC differentially regulates the transcription of these genes. For example, the TSC2 protein level in the MYC-deficient rat fibroblast HO15 cells is increased, whereas that of RPS6 is reduced. MYC has been shown to repress the transcription of *TSC2* and thereby activates the mTORC1/S6K pathway, but the involvement of MYC in the transcriptional regulation of the *RPS6* gene has not been determined yet [31].

Zinc finger BED domain-containing protein 1 (ZBED1), an E3 SUMO-protein ligase, has been reported to bind to the upstream regions of RP genes, including *RPS6* [32]. Depletion of *ZBED1* reduces the transcription of the *RPS6*, *RPSL10A*, and *RPL12* genes. The expression of the *ZBED1* and RP genes is coordinately regulated via a cell-cycle-dependent manner. ZBED1 SUMOylates promoter-bound chromodomain-helicase-DNA-binding protein 3 (CHD3) to release it from the DNA and thereby increases the recruitment of RNA polymerase II to RP gene promoters [33].

Dual-specific tyrosine-phosphorylation-regulated kinase 1A (DYRK1A) has been reported to be recruited to the promoters of genes transcribed by RNA polymerase II [34]. In fact, DYRK1A is constitutively recruited to the promoters of cell-cycle-dependent genes, such as *CDK12*, *RPS6*, *RPS11*, and *RPS12*, and depletion of *DYRK1A* downregulates expression of these genes.

The *RPS6* gene is transcribed in the mid-to-late G_1_ phase, whereas rRNA genes are transcribed in the early G_1_ phase concurrently with the nucleolar assembly [35]. In contrast to the *RPS27a* gene promoter, the human *RPS6* gene promoter is not occupied by SP1 or the cAMP-responsive element-binding protein (CREB) [35]. Interestingly, E2F transcription factor 1 (E2F1) has been detected to bind to the *RPS6* gene promoter [35]. Since E2F1 is a well-established transcription factor that regulates the cell-cycle-dependent transcription [36], it is highly plausible that E2F1 is a key transcriptional regulator of *RPS6* during cell cycle progression. Additionally, the retinoblastoma-associated protein (RB) might contribute to the transcriptional upregulation of *RPS6* by binding to E2F1 on the *RPS6* promoter. *RPS6*-knockdown (KD) is known to downregulate phospho (p)-RB in various cancer cells [37,38,39,40]; however, it is elusive whether such a positive feedback loop between RPS6 and the p-RB level is involved in cancer progression. Furthermore, the precise roles and effects of E2F1 and RB on the transcription of *RPS6* remain to be determined.

### 2.2. Post-Translational Modifications of RPS6

**Figure 1:**
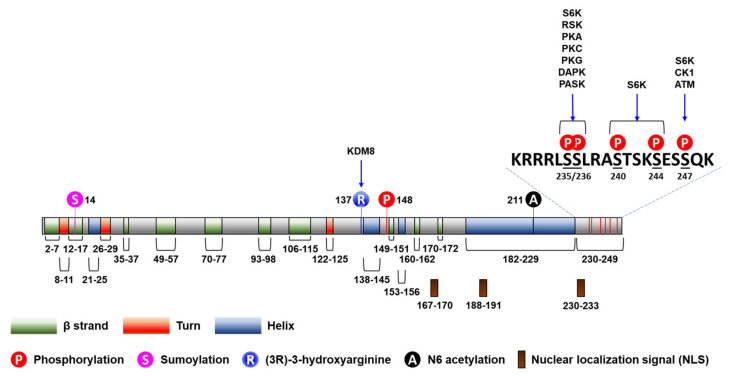
Secondary structure and post–translational modification of RPS6. The structural information was adopted from UniProt Knowledgebase entry P62753 (RS6_HUMAN).

Post-translational modifications, including phosphorylation, acetylation, methylation, *O*-linked β-*N*-acetylglucosaminylation, and ubiquitinylation, have been described to participate in the regulation of the activity of RPs [41] (Figure 1). For example, ischemic preconditioning (IPC) induces *S*-nitrosylation of RPs, including RPS6, in perfused mouse hearts [42]. In addition, the R137 residue of RPS6 has been identified to be hydroxylated by lysine demethylase 8 (KDM8), an L-arginine (3R)-hydroxylase [43]. However, little is known about the regulation of the post-translational modifications of RPS6, except for phosphorylations, which we will discuss next.

#### 2.2.1. Phosphorylation of RPS6

Translation is strictly regulated by signaling pathways sensing environmental stresses (e.g., heat shock or ultraviolet [UV] irradiation), extracellular stimuli (e.g., hormones or growth factors), and intracellular cues (e.g., nutrients, metabolites, energy status, or oxygen availability) [44]. The phosphoinositide 3-kinase (PI3K)/AKT and the mitogen-activated protein kinase (MAPK) pathways are two major pathways involved in the regulation of translation.

RPS6 has five evolutionarily conserved C-terminal serine residues (S235, S236, S240, S244, and S247) that are phosphorylated by various protein kinases (Figure 1) [12,45]. S6 kinase 1 (S6K1) sequentially phosphorylates all these residues, starting with S236 followed by S235, S240, S244, and S247 [46,47]. S235/236 can be also phosphorylated by ribosomal protein S6 kinases (RSKs) [48,49], protein kinase A (PKA; aka cAMP-dependent protein kinase) [50,51,52,53], protein kinase C (PKC) [54], protein kinase G (PKG) [52], and death-associated protein kinase 1 (DAPK1) [55]. S247 can be also phosphorylated by casein kinase 1 (CK1) [56] and ataxia telangiectasia mutated (ATM) [57]. RPS6 is also phosphorylated at S235/236 by PAS domain-containing serine/threonine-protein kinase (PASK), which regulates translation and glycogen synthesis in mammalian cells [58]. Accordingly, p-RPS6 (S240/244) is a more specific readout for S6K activation than the other RPS6 phosphorylation. Interestingly, all these phosphorylated serine residues are dephophorylated by a single phosphatase, protein phosphatase 1 (PP1) [56,59].

**Figure 2:**
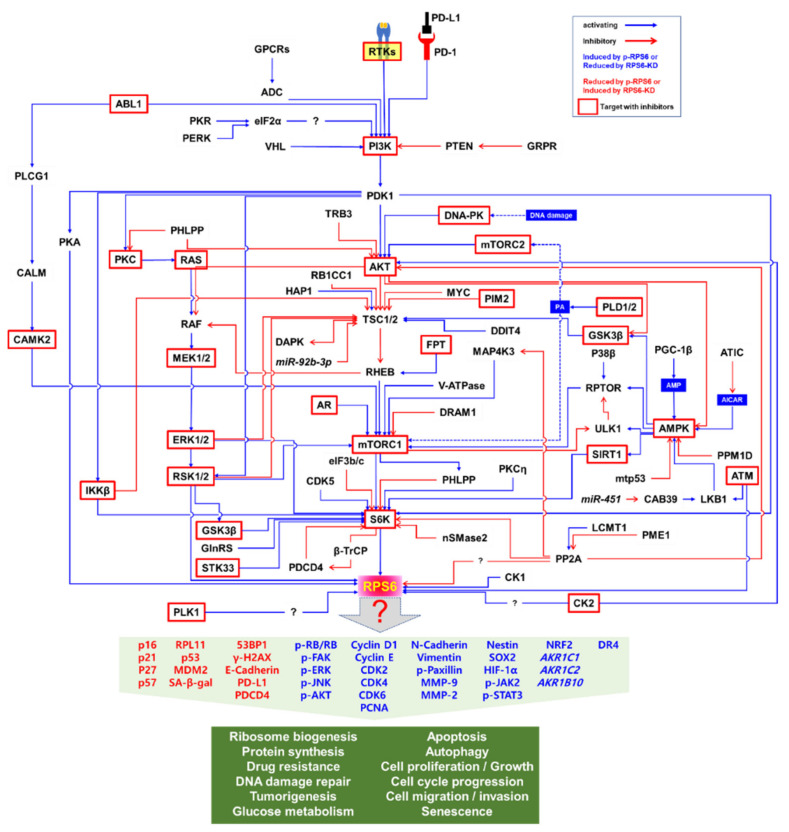
Schematic diagram of the RPS6 regulatory pathway. Arrows indicate unidirectional regulation.

The PI3K/AKT/mTORC1/S6K pathway is the major signaling pathway that upregulates the phosphorylation of RPS6 in mammalian cells (Figure 2) [2]. RPS6 is the first identified substrate of S6K1 (p70S6Kα) [60], and phosphorylation of RPS6 has been used as a readout of mTORC1 activity [2,18,19,20]. However, a close homolog of S6K1, S6K2 (p70S6Kβ), is the major kinase of RPS6 [61,62,63,64,65]. The two S6K homologs S6K1 (S6Kα) and S6K2 (S6Kβ) in mammal share extensive but dispersed homology (43%, 84%, and 59% in N-terminus, catalytic domain, and C-terminus, respectively) [63]. S6K1 has cytosolic (p70S6K1) and nuclear (p85S6K1) isoforms [66], whereas the two isoforms of S6K2 (p54S6K2 and p56S6K2) are nuclear because of a C-terminal nuclear localization sequence (NLS) they have [61,63]. The activity of S6K is tightly controlled via rapid dephosphorylation by pleckstrin homology domain leucine-rich repeat protein phosphates (PHLPP), which is upregulated by mTORC1 [67]. However, the details of the fine regulation of RPS6 phosphorylation by these S6K isoforms are elusive.

The MAPK pathway consists of rat sarcoma (RAS)/v-raf-1 murine leukemia viral oncogene homolog (RAF)/MAPK-ERK kinase (MEK)/extracellular signal-regulated kinase (ERK) cascade. RSKs are downstream effectors of the MAPK pathway, which also regulates RPS6 activity through RPS6 phosphorylation [48]. Four RSK genes, namely *RSK1*—*4*, have been identified in mammals, and these four genes display high homology (78–90%), especially in two kinase domains [68]. In cells lacking S6K, phosphorylation of RPS6 at S235/236 is mediated by RSKs [69]. In resting cells, RSKs are mainly localized in the cytoplasm [70]. Upon stimulation of cells with a growth factor, RSK1 shows maximal kinase activity and transiently translocates to the plasma membrane and then to the nucleus [71]. Subcellular localization of RSK2 is differentially regulated. For example, upon mitogen stimulation of cells, RSK2 localizes to the nucleus; whereas oxidative stress induces RSK2 localization to the cytoplasmic stress granules to promote the survival of the cells [72].

Since S6K and RSK are the downstream effectors of the PI3K/AKT/mTORC1 and MAPK pathways, respectively, RPS6 constitutes a convergence node of these two signaling pathways (Figure 2). In addition, synergistic crosstalk between the mTORC1 and MAPK signaling in the regulation of RPS6 phosphorylation has been reported (see below). ERK enhances S6K activity by phosphorylating it at T421/424 [73]. Additionally, ERK phosphorylates and thereby inhibits tuberous sclerosis 2 (TSC2), consequently leading to mTORC1 activation [74]. ERK also indirectly activates mTORC1 through RSK-mediated phosphorylation of the regulatory-associated protein of mTOR (RPTOR) [75]. RPS6 is also phosphorylated in hippocampal neurons by S6K, which is activated by cyclin-dependent kinase 5 (CDK5) in these cells [76]. Knocking-in the gene of a non-phosphorylatable RPS6 (rpS6^P−/−^) mutant protein resulted in small cell size, hypoinsulinemia, decreased β cell size, and muscle weakness in mice [77,78]. However, the exact roles of RPS6 phosphorylations still remain enigmatic.

##### Upstream Effectors of RPS6 Phosphorylation

Various factors regulating the phosphorylation of RPS6 have been identified, including extracellular proteins, kinases and phosphatases, membrane receptors, transcription factors, and adaptor proteins. For example, a genome-scale siRNA screening against 21,121 genes revealed multiple regulators for p-RPS6 (S235/236) in the pancreatic cancer cell line MIA PaCa-2, which contains a constitutively high p-RPS6 level, followed by confirmation in *TSC1*-null mouse embryonic fibroblasts (MEFs) [79]. Of the 1046 identified candidate regulators of p-RPS6 (S235/236), 632 were validated via individual RNAi. However, most of these p-RPS6 regulators should be further experimentally validated.

(1) Extracellular proteins that regulate RPS6 phosphorylation

Various extracellular proteins such as cytokines and growth factors have been identified as regulators of RPS6 phosphorylation (Table 1). Notably, mitogens, including epidermal growth factor (EGF) and insulin, are well-established activators of RPS6 phosphorylation. However, spatiotemporal fine regulation of RPS6 phosphorylation by multiple extracellular proteins still remains to be elucidated.

(2) Kinases and phosphatases that regulate RPS6 phosphorylation

The human genome is known to encode 656 kinases and approximately 184 phosphatases [97]. Multiple kinases and phosphatases contribute to fine regulation of RPS6 phosphorylation (Table 2). The upstream regulators of RPS6 are controlled in a context- and/or cell-type-dependent manner. In addition, the spatiotemporal regulation is more complex than the simple snapshot illustrated in Figure 2. For example, the activity of AKT is generally regulated by PI3K in response to growth factors, leading to activation of the mTORC1/S6K/RPS6 signaling by inhibiting TSC2 [98,99]. However, DNA damage induces AKT activation via the DNA-dependent protein kinase (DNA-PK) and activated AKT suppresses the RAF/MEK/ERK/S6K1 signaling, thereby reducing the p-RPS6 (S235/236) level, independently of TSC2, mTORC1, or p53 [100]. Under these conditions, the DNA damage-inducible transcript 4 protein (DDIT4)/regulated in development and DNA damage responses 1 (REDD1) inhibits the AKT signal to mTORC1 by sequestering the 14-3-3 protein to activate TSC. In addition, the interaction of Sestrin-2 with the 5′-AMP-activated protein kinase (AMPK) inhibits the AKT-mediated inhibition of AMPK upon DNA damage. Therefore, DNA damage downregulates p-RPS6 (S235/236) by inhibiting the RAF/MEK/ERK/S6K1 signaling through DNA-PK-activated AKT even in the presence of growth factors. These results suggest that changes in p-RPS6 level that are induced by drugs or other stimuli do not always reflect changes in the mTORC1 signaling [100]. The downstream effectors of mTORC1, S6K1, and eukaryotic translation initiation factor 4E-binding protein 1 (4E-BP1) have been also reported to be differentially regulated by exogenous signals. For example, DNA damage suppresses S6K1-mediated RPS6 phosphorylation in a DNA-PK/AKT-dependent manner, whereas p-4E-BP1 does in a p53 and p63 activity-dependent manner [100].

AMPK both negatively and positively regulates p-RPS6. Activated AMPK has been reported to phosphorylate TSC2, which inhibits mTORC1 activity to downregulate p-RPS6 (S240//244) [96]. Conversely, in the TNBC cell line MDA-MB-231, it has been demonstrated that overexpression of peroxisome proliferator-activated receptor-γ (PPAR- γ) coactivator-1β (PGC-1β) increases the concentration of AMP to activate AMPK, which then induces p-RPTOR (S792) and p-RPS6 (S240/244) [101].

Regulation of p-RPS6 by glycogen synthase kinase-3β (GSK3β) is also complex. AMPK-priming phosphorylated GSK3β activates TSC2, leading to downregulation of p-RPS6 (S240/244) [96]. However, GSK3β also induces p-RPS6 through the activation of S6K1 by directly phosphorylating S6K1 at S371 residue in HEK293 cells in response to insulin [83].

**Table 2:** Selected examples of the kinases and phosphatases regulating RPS6 phosphorylation.

Protein	Effects
ABL1 (Abelson murine leukemia viral oncogene homolog 1)	✓ BCR-ABL phosphorylates p-RPS6 (S235/236, S240/244) in chronic myeloid leukemia (CML) cells via mTOR activation [86,102,103,104]
AKT	✓ activates mTORC1 by inhibiting TSC1/2, leading to activation of S6K [98,99] ✓ induces dephosphorylation of p-RPS6 (S235/236) via the RAF/MEK/ERK/S6K1 signaling in a DNA-PK dependent but PI3K-independent manner upon DNA damage [100]
AMPK (AMP-activated protein kinase)	✓ reduces p-RPS6 (S240/244) by inhibiting mTORC1 through TSC2 phosphorylation [96] ✓ a constitutively active mutant AMPKα reduces p-RPS6 (S235/236) in the colorectal cancer (CRC) cell line HT-29 [105] ✓ activated by PGC-1β overexpression, leading to upregulation of p-RPTOR (S792) and p-RPS6 (S240/244) in MDA-MB-231 cells [101]
CK2 (casein kinase 2)	✓ a constitutively active serine/threonine protein kinase composed of two catalytic (α and/or α’) and two regulatory (β) subunits [106,107,108] ✓ up-regulated in imatinib-resistant CML cells [109] ✓ Knockdown of α, α’, or β subunit reduces p-RPS6 (S235/236) but not p-AKT (T308/S473) or p-p70S6K (T389) in imatinib-resistant CML cells [109] ✓ Knockdown of *CSNK2A1* (the gene encoding CK2α) or *CSNK2B* (the gene encoding CK2β) reduces p-RPS6 (S235/236) in an AKT/mTORC1/S6K1-dependent manner in the normal renal tubular epithelial HK-2 cells [110]
DAPK1 (death-associated protein kinase 1)	✓ reciprocal inhibition with TSC2 [111]
DNA-PK (DNA-dependent protein kinase)	✓ reduces p-RPS6 (S235/236) by inhibiting the RAF/MEK/ERK pathway through the DNA-PK/AKT axis in response to DNA damage in p53-deficient cells and tumors [100]
ERK1/2 (extracellular signal-regulated kinase 1/2)	✓ induces p-RPS6 (S240/244) by activating S6K in response to FGFR4 in TNBC MDA-MB-453 cells [112] ✓ induces p-RPS6 (S240/244) in an *HRAS*^G12V^ head and neck squamous cell carcinoma (HNSCC) cell line by blocking TSC2 to activate the mTORC1 signaling [113]
FGFR4 (fibroblast growth factor receptor 4)	✓ induces p-RPS6 (S240/244) via the ERK1/2-S6K axis in MDA-MB-453 cells [112]
GSK3β (glycogen synthase kinase-3β)	✓ inhibits p-RPS6 (S240/244) by activating TSC2 via a mechanism dependent on AMPK-priming phosphorylation [96] ✓ activated by de-phosphorylation in a 2-DG-dependent manner in glycolysis-dependent cancer cell lines [114] ✓ induces p-RPS6 through activating S6K1 by directly phosphorylating its S371 residue in HEK293 cells in response to insulin [83]
IKKβ (inhibitor of NF-κB kinase β)	✓ activates p85S6K1, but not p70S6K1, by physical association in response to H_2_O_2_ in a PI3K/mTOR-independent manner in MCF7 cells. However, the role of IKKβ/p85S6K1/p-RPS6 in the H_2_O_2_-induced cell death remains unknown [115]. ✓ represses TSC1 by interacting with and phosphorylates TSC1 at S487 and S511, leading to activation of the mTOR pathway [95]
MAP4K3 (mitogen-activated protein kinase kinase kinase kinase 3)	✓ also known as germinal center kinase-like kinase (GLK) ✓ induces p-RPS (S240/244) through the mTORC1-S6K axis in response to amino acids [116] ✓ *MAP4K3*-KD reduces p-RPS6 (S235/236) in MIA PaCa-2 cells [79] ✓ dephosphorylated by PP2A at S170 in response to amino acids, leading to dephosphorylation of p-RPS6 (S240/244) through the MAP4K3/mTORC1/S6K pathway [117]
mTOR	✓ *MTOR*-KD reduces p-RPS6 in bladder cancer cells [118]
P38β	✓ activates mTORC1 by phosphorylating RPTOR at S863/S771 in response to arsenite [119] ✓ identified as a p-RPS6 (S235/236) regulator in MIA PaCa-2 cells [79]
PDK1 (3-phosphoinositide-dependent protein kinase 1)	✓ activates AKT [120,121], p70S6K [122], p90RSKs [123], protein kinase A (PKA) [124], protein kinase C delta (PKCδ), PKCζ [125], serum and glucocorticoid-inducible kinases (SGKs), p21-activated kinase (PAK1) [126], serine/threonine-protein kinase N1/2 (PKN1/2) [127], and IKKβ [128] ✓ induces p-RPS6 through activation of p70S6K by directly phosphorylating p70S6K at T252 independently of phosphatidylinositol 3,4,5-triphosphate [PtdIns(3,4,5)P_3_] [129]
PERK (PRKR-like endoplasmic reticulum kinase)	✓ induces p-RPS6 (S235/236) through the PI3K/AKT/mTOR pathway in a eukaryotic translation initiation factor 2α (eIF2α) S51 phosphorylation-dependent manner in response to ER stress (thapsigargin) [130]
PI3K p110α (phosphoinositide 3-kinase 110kDa catalytic subunit α)	✓ Knockdown of *PIK3CA* (the gene encoding p110α), but not *PIK3CB* (the gene encoding p110β), reduces p-RPS6 (S235/236 and S240/244) and p-AKT (S473) in the small cell lung cancer cell (SCLC) line, H69 and concomitantly reduces the cell viability [131]
PIM2 (serine/threonine-protein kinase PIM2)	✓ induces p-RPS6 (S235/236) by inhibiting TSC2 via TSC2 phosphorylation at S1798 in multiple myeloma (MM) cells [132] ✓ identified as a p-RPS6 (S235/236) regulator in MIA PaCa-2 cells [79] ✓ Knockdown of *PIM2*, but not *PIM1*, reduces p-RPS6 (S235/236) and p-p70S6K (T389) in glioblastoma multiforme (GBM) LN229 cells [133]
PKA (protein kinase A; also known as cyclic AMP-dependent protein kinase)	✓ phosphorylates S235/236 of RPS6 [50,51,52,53]
PKR (protein kinase RNA-activated)	✓ induces p-RPS6 (S235/236) through the PI3K/AKT/mTOR pathway in response to double-stranded RNAs [130]
PHLPP1/2 (PH domain leucine-rich repeat-containing protein phosphatase 1/2)	✓ a tumor suppressor that negatively regulates AKT and S6K1 [134], and PKC [135] through direct dephosphorylation ✓ Loss of PHLPP upregulates p-RPS6 (S235/236) and the amount of p-RPS6 bound to the translation initiation complex via activating S6K1 in response to insulin, leading to increases in cell size, protein content, and rate of cap-dependent translation [134] ✓ activated by mTORC1 in response to glucose or amino acids in colon and breast cancer cells [67]
PLK1 (Polo-like kinase 1)	✓ *PLK1*-KD downregulates p-RPS6 (S235/236), whereas active PLK1 induces p-RPS6 (S235/236) [136]
PP2A (serine/threonine-protein phosphatase-2A)	✓ downregulates p-RPS6 (S235/236) by inactivating both AKT and S6K via a mechanism dependent on leucine carboxyl methyltransferase 1 (LCMT1)-mediated methylation [137] ✓ dephosphorylates p-RPS6 to block translation via an unknown mechanism independent of S6K [137] ✓ dephosphorylates p-MAP4K3 (S170) to inhibit the MAP4K3/mTORC1/S6K pathway, leading to dephosphorylation of p-RPS6 (S240/244) [117]
PPM1D/WIP1 (protein phosphatase magnesium-dependent 1 delta/wild-type p53-induced phosphatase)	✓ induces p-RPS6 (S235/236) through the AMPK/mTORC1 pathway by dephosphorylating p-AMPKα (T172) independently of AKT, leading to proliferation and neointima hyperplasia after vascular injury, in the mouse [92]
PTEN (phosphatase and tensin homolog)	✓ a tumor suppressor, acting as a dual-specificity protein phosphatase that dephosphorylates tyrosine-, serine- and threonine-phosphorylated proteins [138] ✓ Loss of *PTEN* resulted in upregulation of p-RPS6 (S235/236) through activation of the AKT/mTOR pathway [139] ✓ *Pten*-KD induces p-RPS6 (S240/244) in rat cortical neuron [140]
RACK1 (receptor of activated protein kinase C kinase 1)	✓ *RACK1*-KD downregulates p-RPS6 (S235/236), p-AKT (S473), and p-mTOR (S2448) in the oral squamous cell carcinoma (OSCC) cell line HSC-3 in vitro and in OSCC xenografted tumors in vivo [141]
RSK1/2 (ribosomal protein S6 kinase 1/2)	✓ activates the mTORC1/S6K/RPS6 pathway by phosphorylating TSC2, RPTOR, and RPS6 in response to serum, PMA, or EGF [48,75,142]
S6K1 (ribosomal protein S6 kinase beta-1)	✓ induces p-RPS6 (S235/236, S240/244) in response to serum [48] ✓ Mutations (S371A, T389A, and K100R) abolish p-RPS6 in response to insulin, PMA, and serum [83]
STK33 (serine/threonine kinase 33)	✓ *STK33*-KD reduces p-RPS6 (S235) in an S6K-dependent manner in the human SCLC cell line NCI-H446 both in vitro and in xenografted tumors [143]
ULK1 (Unc-51-like kinase 1)	✓ a serine/threonine kinase that promotes autophagy in response to starvation [144] ✓ inhibits mTORC1 by interacting with and phosphorylating RPTOR, leading to a reduction in p-RPS6 (S235/236) [145] ✓ phosphorylated and activated by AMPK in response to nutrient starvation [146] ✓ phosphorylated and inactivated by mTOC1 in response to nutrient [147]

(3) Membrane proteins that regulate RPS6 phosphorylation

Beyond receptor tyrosine kinases, membrane proteins that regulate phosphorylation of RPS6 have also been studied (Table 3). Interestingly, the programmed cell death protein 1 (PD-1)/programmed death ligand 1 (PD-L1) pathway has been found associated with p-RPS6. PD-1 binds to both RPS6 and eIF-4E to promote their phosphorylation in a hepatocellular carcinoma (HCC) cell line [148]. In addition, a recombinant PD-L1 fragment crystallizable (Fc) fusion protein induces p-RPS6 in a murine melanoma cell line in an mTOR-dependent, but not PI3K-dependent manner [149]. In contrast, PD-L1 expression has been reported to be negatively correlated with the p-RPS6 (S235/236) level in non-small cell lung cancer (NSCLC) clinical samples [150]. Since *RPS6*-KD induced PD-L1 expression and immune-resistance in breast and prostate cancer cells [151], further studies will clarify the potential role of p-RPS6 in immuno-oncological regulation.

(4) Transcription factors that regulate RPS6 phosphorylation

Transcription factors have also been reported to regulate RPS6 phosphorylation via various mechanisms (Table 4). The Forkhead box protein O17 (FOXO17), MYC, and mutant (mt) p53^P151S^ have been reported to induce p-RPS6 indirectly [30,153,154]. MYC transcriptionally suppressed *TSC2* gene expression to activate S6K kinase activity, leading to an increase in p-RPS6 [30]. In addition, mtp53 blocked the AMPK activity through direct binding to the AMPKα subunit, leading to upregulation of p-RPS6 (S240/244) in head and neck squamous cell carcinoma (HNSCC) cells [154]. In contrast, androgen receptor (AR), basic transcription factor 3 (BTF3), and FOXO3 negatively regulated p-RPS6. The mechanism of AR- and BTF3-mediated suppression remains to be determined [155,156], whereas FOXO3 has been reported to activate *TSC1* transcription [157].

(5) Other proteins that regulate RPS6 phosphorylation

In addition to the above-mentioned factors, various proteins have also been reported to regulate RPS6 phosphorylation (Table 5). Notably, DNA damage-related proteins, such as DNA damage-binding protein 1 (DDB1) and DDIT4, contribute to the regulation of RPS6 phosphorylation. Mechanistically, DDIT4 negatively regulates p-RPS6 by activating TSC function [100,158].

It has also been reported that tumor suppressor proteins negatively regulate p-RPS6. Programmed cell death 4 (PDCD4) was reported to downregulate p70S6K1 phosphorylation and translation, but not p70S6K2, leading to chemosensitivity of colorectal cancer (CRC) cells to the insulin-like growth factor 1 receptor (IGF1R) inhibitor linsitinib (OSI-906) [103]. Interestingly, the expression of PDCD4 is negatively regulated by p70S6K1-mediated phosphorylation and the subsequent proteasomal degradation mediated by beta-transducin repeat containing protein (β-TrCP) [159]. In contrast, von Hippel–Lindau disease tumor suppressor (VHL) activates the PI3K/AKT/mTOR/S6K pathway by direct binding to PI3K p110, thereby upregulating p-RPS6 (S235/236) in HEK293 cells [160].

**Table 5:** Selected examples of the other proteins regulating RPS6 phosphorylation.

Protein	Effects
ADAR1 (adenosine deaminase acting on RNA 1)	✓ an enzyme catalyzes A-to-I RNA editing (deamination of adenosine to inosine in double-stranded RNA) [161] ✓ *ADAR1*-KD induces cell death and growth arrest in vitro, downregulates p-RPS6 (S235/236), p-mTOR (S2448), and p-p70S6K (T389) in gastric cancer (GC) cells, and attenuates tumorigenicity and metastasis of GC cells in vivo [162] ✓ Overexpression of ADAR1 increases p-RPS6 (S235/236), p-mTOR (S2448), and p-p70S6K (T389) in GC cells
AMOG (adhesion molecule in glia)	✓ induced p-RPS6 (S240/244) by activating the AKT/mTOR/S6K pathway, leading to increase in cell size, in a TSC/RHEB-independent manner [163]
ATIC (AICAR transformylase/inosine monophosphate cyclohydrolase)	✓ the last enzyme of de novo purine synthesis, which transfers a formyl group to AICAR to produce the intermediate formyl-AICAR and inositol monophosphate (IMP) [164] ✓ *ATIC*-KD inhibits p-RPS6 (S235/236), p-p70S6K (T389), and p-mTOR (S2448) through activation of AMPK in hepatocellular carcinoma cell lines [165]
DDB1 (DNA damage-binding protein 1)	✓ *DDB1*-KD inhibits p-RPS6 (S240/244) in MIA PaCa-2 cells [79]
DDIT4/REDD1 (DNA damage-inducible transcript 4 protein/regulated in development and DNA damage responses 1)	✓ acts upstream of the TSC1/2 complex to suppress p-RPS6 (S235/236) in response to hypoxia but not to serum in MEFs [158] ✓ sequesters 14-3-3 to activate TSC function to block AKT signaling in response to DNA damage [100]
DRAM1 (DNA damage-regulated autophagy modulator protein 1)	✓ Overexpression of DRAM1 downregulates p-RPS6 (S235/236 and S240/244) via in an mTORC1-dependent manner in HEK293T cells [166]
eIF3 (eukaryotic translation initiation factor 3)	✓ a large (~800 kDa) complex composed of 13 subunits (eIF3a—eIF3m) [167,168] ✓ overexpression of eIF3a, eIF3b, eIF3c, eIF3h, eIF3i, and eIF3m has been found in many cancers [169] ✓ eIF3b and eIF3c interact with inactive S6K1. Mitogen stimuli promote eIF3s binding to mTOR/RPTOR and activation of S6K1 by phosphorylation and subsequent dissociation [170] ✓ *EIF3A*-KD and/or *EIF3C*-KD decreases proliferation and cell size of normal human fibroblast IMR90 with a concomitant increase in p-RPS6 (S240/244) through activation of S6K1 [171]
ETV6/RUNX1 (E/R) fusion protein	✓ induces p-RPS6 through the PI3K/AKT/mTOR pathway activation in E/R-positive B-cell precursor (BCP) acute lymphoblastic leukemia (ALL) cells [172]
FASN (fatty acid synthase)	✓ *FASN*-KD induces apoptosis and downregulates both p-RPS6 (S240/244) and total (t)-RPS6 through AKT/mTOR/SK6 axis by stimulating protein ubiquitination in ovarian cancer cells [173]
Gal-13/PLAC8 (galectin-13/placenta-specific 8)	✓ absent in healthy adult exocrine pancreas but ectopically expressed in pancreatic ductal adenocarcinoma [174,175,176] ✓ *PLAC8*-KD downregulates p-RPS6 (S235/236) and p-RB (S807/811) in human BON-1 pancreatic neuroendocrine tumor cells [177]
GlnRS/QARS (glutaminyl-tRNA synthase)	✓ *GlnRS/QARS*-KD by RNAi inhibits p-RPS6 (S235/236) in MIA PaCa-2 cells [79] ✓ Depletion of *GlnRS/QARS* leads to inhibition of protein synthesis and S6K activation by phosphorylation at T389 [79]
HAP1 (huntingtin-associated protein 1)	✓ inhibits p-RPS6 (S235/236) by suppressing mTORC1 activity through interaction with TSC1 to upregulate its abundance in primary hippocampal neurons [178]
IKAP (IκB kinase complex-associated protein)	✓ *IKAP*-KD reduces p-RPS6 (S235/236) concurrently with an increase in cell growth in LNCaP prostate cancer cells [155] ✓ antagonizes AR-dependent proliferation in LNCaP cells via both mTORC1-dependent and mTORC1-independent pathways [155]
JAK2 (Janus kinase 2)	✓ *JAK2*-KD increases cell proliferation with concordant increases in p-p70S6K (T389), p-4E-BP1 (T37/46), and p-RPS6 (S235/236) in LNCaP prostate cancer cells [155]
LCMT1 (leucine carboxyl methyltransferase 1)	✓ downregulates p-RPS6 (S235/236) through inactivating both AKT and S6K by activating PP2A through methylation in anchorage-independent conditions [137] ✓ *LCMT1*-KD increases p-RPS6 (S235/236) by activating AKT and S6K, leading to enhanced anchorage-independent cell growth and transformation, but not anchorage-dependent growth [137]
LPIN1 (lipin 1)	✓ a phosphatidate phosphatase (3-*sn*-phosphatidate phosphohydrolase) that converts phosphatidic acid to diacylglycerol during triglyceride, phosphatidylcholine and phosphatidylethanolamine biosyntheses to control the metabolism of fatty acids [179] ✓ acts as a transcriptional coactivator for PPARα and PGC-1α to control the expression of genes in lipid metabolism [180,181,182] ✓ Depletion of *LPIN1* suppresses proliferation and migration, induces autophagy, and reduces p-AKT (S473) and p-RPS6 (S235/236) in a prostate cancer PC3 cell [183] ✓ Depletion of *LPIN1* sensitizes PC-3 and HS578T to rapamycin [183]
nSMase2 (neutral sphingomyelinase-2)	✓ mediates all-*trans*-retinoic acid (ATRA) effects on an increase in ceramide, G_0_/G_1_ cell-cycle arrest, and suppression of p-RPS6 via inhibiting p-S6K in a breast cancer cell line MCF7 [184] ✓ Overexpression of nSMase2 induces G_0_/G_1_ growth arrest in MCF7 cells [184]
p62/SQSTM (sequestome-1)	✓ an integral part of the mTORC1 complex by interacting with RPTOR to mediate amino acid signaling for S6K1 activation [185] *SQSTM1*-KD inhibits p-RPS6 (S235/236) in MIA PaCa-2 cells [79]
PDCD4 (programmed cell death 4)	✓ a tumor suppressor that inhibits translation initiation and cap-dependent translation [186,187] ✓ upregulated by imatinib or rapamycin in BCR-ABL CML cells [103] ✓ upregulated by nilotinib through both an mTOR/p70S6K-inhibition-mediated blocking of protein degradation and induction of its transcription in BCR-ABL CML cells [103] ✓ confers linsitinib (OSI-906) chemosensitivity to CRC cells by inhibiting the phosphorylation and translation of p70S6K1, but not p70S6K2 [188,189] ✓ negatively regulated by p70S6K1-mediated phosphorylation and subsequent proteasomal degradation in a β-TrCP-dependent manner [159]
PGC-1β (peroxisome proliferator-activated receptor-γ (PPAR- γ) coactivator-1β)	✓ Overexpression of PGC-1β activates AMPK by increasing AMP level, leading to upregulation of p-RPTOR (S792) and p-RPS6 (S240/244), whereas knockdown of *PPARGC1B* (the gene encoding PGC-1β) suppresses AMPK activation in MDA-MB-231 cells [101] ✓ Overexpression of PGC-1β reduces p-RICTOR (T1135) and p-AKT (S473), whereas *PPARGC1B*-KD shows the opposite effect in MDA-MB-231 cells [101]
PLCG1 (phospholipase C-γ1)	✓ induces p-RPS6 (S235/236 and S240/244) through the BCL-ABL/PLCG1/Calmodulin (CALM)/CALM kinase (CAMK)/mTORC1/S6K pathway in parallel with the PI3K/AKT/mTORC1/S6K pathway in CML cells [104]
PME1 (protein phosphatase methylesterase 1)	✓ upregulates p-RPS6 (S235/236) through activating the AKT/S6K pathway by inactivating PP2A through demethylation in anchorage-independent conditions [137]
RAS	✓ induces p-RPS6 (S235/236 and S240/244) in a MEK-dependent mechanism [48] ✓ HRAS^G12V^ induces p-RPS6 (S240/244) in an HNSCC cell line through the ERK-TSC2 signaling [113]
RB1CC1 /FIP200 (RB1-inducible coiled-coil protein 1/FAK family kinase-interacting protein of 200 kDa)	✓ induces p-RPS6 (S240/244) through the TSC1/TSC2-S6K axis in response to nutrients, increasing cell size but not cell proliferation [190]
RHEB (RAS homolog enriched in brain)	✓ induces p-RPS6 (S235/236) through the mTORC1-S6K axis, whereas inhibits BRAF activity in rat ELT3 cells [191] ✓ *RHEB*-KD downregulates p-RPS6 (S235/236) in MCF7 cells [115] ✓ enhances mTORC1 substrate recognition via increasing RPTOR binding [192,193] ✓ restores 2-DG-ablated p-RPS6 (S235/236 and S240/244) and p-p70S6K (T389) in a glycolysis-dependent cancer cell line [114]
SHE1 (nucleoporin SHE1)	✓ A component of GAP activity towards RAGs 2 (GATOR2) complex that indirectly activates mTORC1 through inhibiting the GATOR1 complex [194] ✓ *SHE1*-KD by RNAi inhibits p-RPS6 (S235/236) in MIA PaCa-2 cells [79]
SIRT1 (NAD-dependent protein deacetylase sirtuin-1)	✓ induced p-RPS6 (S235/236) by deacetylating S6K1, leading to enhancement of S6K1 phosphorylation by mTORC1 in gut adult stem cells in response to calorie restriction [195] ✓ SIRT1 activators repress p-RPS6 (S235/236) via an unknown mechanism [196]
TRB3 (Tribbles homolog 3)	✓ An endogenous inhibitor of AKT [197] ✓ reduces insulin-induced p-RPS6 (S235/236) through the AKT/mTOR/S6K axis both in vitro and in vivo [198]
TSC1 (Tuberous sclerosis 1 protein)	✓ *Tsc1*-KD upregulates p-RPS6 (S235/236) in carotid arteries and VSMCs isolated from *Tsc1^KD^* mice [92]
TSC2 (Tuberous sclerosis 2 protein)	✓ downregulates p-RPS6 (S235/236) via mTOR in response to hypoxia in MEFs [158] ✓ downregulates p-RPS6 (S240/244) via S6K1 in response to serum-starvation in MEFs [199]
URM1 (ubiquitin-related modifier 1)	✓ *URM1*-KD inhibits p-RPS6 (S240/244) in MIA PaCa-2 cells [79]
V-ATPase (vacuolar proton-ATPase)	✓ transmits an activating signal, that is generated by accumulated amino acids inside the lysosomal lumen, to the RAG GTPases to recruit mTORC1 to the lysosomal surface [200] ✓ Knockdown inhibits p-RPS6 (S235/236) in MIA PaCa-2 cells [79]
VHL (von Hippel-Lindau disease tumor suppressor)	✓ a tumor suppressor possessing a ubiquitin ligase activity and also serving as an adaptor protein for signaling regulators in a ubiquitin ligase activity-independent manner [201,202] ✓ activates the PI3K/AKT/mTOR/S6K pathway by direct binding to PI3K p110 [160] ✓ *VHL*-KD downregulates p-RPS6 (S235/236) in HEK293 cells [160]

##### MicroRNAs That Regulate RPS6 Level

Only a few microRNAs (miRNAs) have been identified as regulators of RPS6 expression (Table 6). MiR-92b-3p and miR-451 have been reported to target *TSC1* [203] and calcium-binding protein 39 (*CAB39*) [204], respectively, to activate mTORC1. In addition, miR-451 further activated the AMPK/mTOR pathway to induce p-RPS6 (S235/235) in CRC cells via an unknown mechanism [105]. Further studies will be needed to identify additional miRNAs involved in the regulation of RPS6.

##### Natural Ligands or Stimuli That Regulate RPS6 Phosphorylation

Endogenous natural ligands and stimuli also regulate RPS6 phosphorylation (Table 7). Mitogenic ligands or stimuli including amino acids, all-*trans-*retinoic acid (ATRA), 5α-dihydrotestosterone (DHT; androgen), and 17-β estradiol (E_2_) induce p-RPS6, whereas alpha-linoleic acid (ALA), hypoxia, mannitol, and oleic acid (OA) downregulate p-RPS6. The effect of hydrogen peroxide is controversial. Hydrogen peroxide has been shown to induce p-RPS6 (S235/236) in MCF7 and HCT116 cells in an mTOR-independent manner (Jia et al., 2013), but it has also been shown to downregulate p-RPS6 (S235/235) in the cytoplasm of MCF7 cells through a mechanism involving ATM/liver kinase B1 (LKB1)/AMPK/TSC2 [205]. The detailed mechanism of this differential regulation remains elusive.

##### Pharmacological Perturbation of the Upstream Effectors of RPS6

To date, various agents including antibodies and small molecules have been reported to perturbate the upstream effectors of RPS6 (Table 8). Antibodies against cytokines, growth factors, or membrane proteins have been reported to modulate RPS6 phosphorylation. For example, the anti-cathepsin L/MEP antibody [81] and the anti-PD-1 monoclonal antibody (mAb) [149] have been reported to inhibit RPS6 phosphorylation. Conversely, IFNα and IFNβ upregulate p-RPS6 (Table 1), and anti-IFNα/β receptor 2 antibody (anti-IFNAR2 Ab) induces p-RPS6 [87].

Notably, many small-molecule protein kinase inhibitors (PKIs) downregulate p-RPS6 by blocking specific protein kinases. Several of these PKIs, such as alpelisib, dabrafenib, everolimus, imatinib, nilotinib, rapamycin, selumatinib, trametinib, and vemurafenib, have been approved by the US Food and Drug Administration (FDA) and clinically applied to treat various diseases including cancers [213].

Unlike AMPK itself [see Section Upstream Effectors of RPS6 Phosphorylation. (2) Kinases and phosphatases that regulate RPS6 phosphorylation], all AMPK activators (5-aminoimidazole-4-carboxamide ribonucleotide (AICAR), metformin, and phenformin) downregulate p-RPS6 [105,214]. Conversely, the AMPK inhibitor dorsomorphin abolishes GSK28030371-mediated downregulation of p-RPS6 in the presence of PDGF-BB [92]. 

Results derived from experiments involving small-molecule PKIs have suggested new protein kinases as regulators of RPS6 phosphorylation. For example, pan-PIM kinase inhibitors such as AZD1208 [215], LGB321 [132], and SGI-1776 [133], have been reported to downregulate p-RPS6. BI2536, an inhibitor of Polo-like kinases (PLKs), also inhibits RPS6 phosphorylation [136]. These results suggest that PIM and PLKs regulate p-RPS6 either directly or indirectly. Other potential upstream regulators of RPS6 phosphorylation, such as STK33 [143] and IKK [115], may also be identified by the applications of kinase-specific PKIs.

Various protein kinases crosstalk with each other, forming both positive and negative feedback and feedforward loops (Figure 2). For example, pharmacological inhibition of mTORC1/S6K suppresses the negative feedback loops leading to the activation of compensatory upstream signaling molecules including AKT [216,217,218]. Notably, targeting mTORC1 with rapalogs as monotherapies to treat tumors has had limited success in hundreds of clinical trials [219]. Therefore, targeting RPS6 and its downstream rather than upstream effectors might be an alternative approach for cancer treatment.

**Table 8:** Selected examples of the agents regulating the upstream of the RPS6 signaling.

Agent	Effects
**Antibodies**	
Anti-cathepsin L/MEP Ab	✓ inhibit C3-induced p-RPS6 and p-S6K1 (S371) in Paneth cells from acute gastrointestinal injury (AGI)-produced mice [81]
Anti-IFNAR2 Ab (Anti-IFNα/β receptor 2 antibody)	✓ induces p-RPS6 (S240/242) at 24 and 48 h post-treatment through activating RPS6KB in Daudi cells [87]
Anti-PD-1 mAb	✓ downregulates p-RPS6 in both B16-F10 murine melanoma and human melanoma in mice with reduced growth of melanoma [149]
**Small molecule inhibitors or activators**	
AICAR (5-aminoimidazole-4-carboxamide ribonucleoside; Acadesine)	✓ an activator of AMPK and AMPK kinase (AMPKK) [220] ✓ decreases p-RPS6 (S235/236) in the CRC cell line HT-29 [105]
Alpelisib (BYL719)	✓ a selective PI3K p110α inhibitor (IC_50_ = 5 nM) [221] ✓ reduces p-RPS6 (S240/244) in HRAS wild-type HNSCC cell lines but not a HRAS^G12V^ mutant cell line due to the ERK/TSC2 signaling [113]
AR-A014418	✓ an inhibitor of GSK3β (IC_50_ = 104 nM) [222] ✓ reduces p-RPS6 by inactivating S6K1 in breast cancer cells, colon cancer, kidney cells, osteosarcoma cells, and prostate cancer cells [83]
Aspirin	✓ inhibits p-RPS6 (S235/235) (S240/244) by reducing mTORC1/S6K signaling either AMPK-dependent or -independent manner in CRC cells, leading to induction of autophagy [223]
AZD1208	✓ a potent inhibitor of PIM1 (IC = 0.4 nM), PIM3 (IC = 1.9 nM), and PIM2 (IC = 5 nM) [224] ✓ downregulates p-RPS6 (S235/236) and p-mTOR (S2448) but upregulates p-eIF2α (S51), p-AMPK (T172), and p-LKB1 (S428) in the human liposarcoma cell line 93T449 [215]
AZD8055	✓ an ATP-competitive mTOR inhibitor (IC = 0.8 nM) [225] ✓ reduces p-RPS6 (S235/236) in normal human dermal fibroblasts (NHDFs) [100] ✓ reduces p-RPS6 (S240/244) in an HRAS^G12V^ mutant cell line by blocking the ERK/TSC2 signaling [113] ✓ reduces p-RPS6 (S235/236) and p-AKT (S473) and induces PD-L1 in cancer cells [150]
BI2536	✓ an inhibitor of Polo-like kinases (PLKs) and bromodomain 4 (BRD4), leading to suppression of MYC [226,227] ✓ reduces p-RPS6 (S235/236) [136]
Bicalutamide (Casodex)	✓ an androgen receptor (AR) antagonist (IC_50_ = 160 nM) ✓ reduces p-RPS6 (S235/236) by inhibiting DHT-stimulated mTORC1 activity in an AR-mediated manner in LNCaP prostate cancer cells [207]
BMS-754807	✓ an inhibitor of insulin receptor (INSR) (IC_50_ = 1.8 nM), IGF1R (IC_50_ = 1.8 nM), TRKB (IC_50_ = 4.1 nM), MET (IC_50_ = 5.6 nM), TRKA (IC_50_ = 7.4 nM), AURKA (IC_50_ = 9 nM), AURKB (IC_50_ = 25 nM), and RON (IC_50_ = 44 nM) [228] ✓ induces p-RPS6 by activating p70S6K1 and MEK1/2 in HCT116 CRC cells [188,189]
BPTES	✓ a selective inhibitor of glutaminase kidney isoform GLS1 (IC_50_ = 0.16 μM) [229] ✓ modestly reduces p-RPS6 (235/236) and p-p70S6K (T389) and synergizes with 2-DG to ablate p-RPS6 (235/236) and p-p70S6K (T389) in glycolysis-independent cancer cell lines [114]
Buparlisib (BKM120, NVP-BKM120)	✓ an inhibitor of PI3K p110α (IC_50_ = 52 nM), p110δ (IC_50_ = 116 nM), p110β (IC_50_ = 166 nM), and p110γ (IC_50_ = 262 nM) [230] ✓ reduces p-RPS6 (S235/236), but not p-AKT (S473), and induces PD-L1 in an NSCLC cell line HCC827 [150]
Butyrate	✓ a short-chain fatty acid produced from anaerobic fermentation of dietary fiber by gut microbiota and inhibits the activity of histone deacetylases (HDACs) [231] ✓ downregulates p-RPS6 (S235/236) via reducing p-S6K1 (T389) and inducing Ac-S6K1 by suppression of SIRT1 protein level in a CCR cell line HCT116 [232]
1-butanol (1-BtOH)	✓ reduces p-RPS6 via the mTOR/S6K axis by inhibiting PLD1/2 in CRC cells [212]
C75 trans (trans-C75, (±)-C75)	✓ a fatty acid synthase (FASN) inhibitor (IC_50_ = 35 μM) [233] ✓ induces apoptosis and downregulates both p-RPS6 (S240/244) and t-RPS6 via AKT/mTOR/SK6 axis by stimulating protein ubiquitination in ovarian cancer cells [173]
Caffeine	✓ inhibits ATM (IC_50_ = 0.2 mM), mTOR (IC_50_ = 0.4 mM), ATR (IC_50_ = 1.1 mM), CHEK1 (IC_50_ = ~5 mM), and DNA-PKcs (IC_50_ = ~10 mM) [234] ✓ reduces p-RPS6 (S235/236) through the PI3K/AKT/mTOR/S6K pathway in neuroblastoma cells [235]
CB-839 (Telaglenastat)	✓ a potent, selective, and orally bioavailable glutaminase inhibitor (IC_50_ = 24 nM), having antitumor activity in TNBC cells [236] ✓ modestly reduces p-RPS6 (235/236) and p-p70S6K (T389) and synergizes with 2-DG to ablate p-RPS6 (235/236) and p-p70S6K (T389) in glycolysis-independent cancer cell lines [114]
Cisplatin	✓ reduces p-RPS6 (S235/236) in MEFs in a DNA-PK-dependent manner [100] ✓ induces p-RPS6 (S235/236) in a cervical cancer cell line HeLa by activating S6K1, leading to cisplatin resistance, but not in a lung cancer cell line SiHa [237]
Cocaine	✓ reduces p-RPS6 (S235/236) in immortalized murine microglial BV-2 cells [238]
Compound 44	✓ an FTL3 inhibitor [239] ✓ reduces p-RPS6 (S235/236 and S240/244) in leukemia cells [240]
CX-5011	✓ an ATP-competitive inhibitor of CK2α (IC50 = 2.3 nM) [241] ✓ reduces p-RPS6 (S235/236) and p-AKT (S129) in imatinib-resistance CML cells without affecting p-BCR-ABL, p-AKT (T308/S473), p-GSK3β (S9), p-p70S6K (T389) and p-ERK1/2 (T202/Y204) [109] ✓ reduces protein synthesis, induces apoptosis, and overcomes the imatinib-resistance in imatinib-resistant CML cells [109]
Cycloheximide	✓ increases p-RPS6 (S240/244) in a normal human fibroblast IMR90 with a reduction of size in G0/G1-, S-, and G2/M-phases [171]
Dabrafenib (GSK2118436)	✓ an inhibitor of BRAF^V600E^ (IC_50_ = 0.7 nM), BRAF (IC_50_ = 5.2 nM), and CRAF (IC_50_ = 6.3 nM) [242] ✓ reduces p-RPS6 in human melanoma cells [243]
Dactolisib (BEZ235)	✓ a PI3K and mTOR inhibitor (IC_50_ value for p110α, p110γ, mTOR (p70S6K), p110δ, ATR, p110β is 4, 5, 6, 7, 21, 75 nM, respectively) [244,245] ✓ reduces p-RPS6 in bladder cancer cells [118] ✓ reduces p-RPS6 (S235/236) and p-AKT (S473) and induces PD-L1 in an NSCLC cell line HCC827 [150]
Deferasirox	✓ an oral iron chelator approved clinically [246] ✓ suppresses p-RPS6 by inducing DDIT4/REDD1 in human myeloid leukemia cells [247]
2-deoxyglucose (2-DG)	✓ a glucose analog widely investigated as a pharmacological agent targeting glycolysis [248] ✓ reduces p-RPS6 (S240/244) by enhancing the interaction between AMPK and TSC2 [96] ✓ reduces p-RPS6 (235/236) and p-p70S6K (T389) only in glycolysis-dependent cancer cell lines [114] ✓ activates AMPK only in glycolysis-dependent cancer cell lines and increases DDIT4/REDD1 [114]
Dexamethasone	✓ a glucocorticoid that induces cell cycle arrest [249] ✓ reduces p-RPS6 (S240/244) in the mouse lymphosarcoma P1798.C7 cells [90]
DG2	✓ a selective S6K inhibitor [250] ✓ reduces p-RPS6 (S240/244) in PC3 cells [251]
Dorsomorphin (Compound C)	✓ a reversible, selective AMPK inhibitor (Ki = 109 nM) ✓ abolishes the GSK28030371-mediated downregulation of p-RPS6 (S235/236) through the AMPK/mTORC1 pathway in the presence of PDGF-BB in vascular smooth muscle cells [92]
Doxorubicin	✓ activates p-RPS6 (S240) via activation of S6K1 in neonatal rat cardiomyocyte, leading to cell death by autophagy [252]
Etoposide	✓ reduces p-RPS6 (S235/236) in NHDFs [100] ✓ reduces p-RPS6 (S235/236) in MEFs in a DNA-PK-dependent manner [100]
Everolimus (RAD001)	✓ an allosteric mTOR inhibitor of FKBP12 (IC_50_ = 1.6–2.4 nM) [253] ✓ reduces p-RPS6 (S235/236 and S240/244) in leukemia cells [104] ✓ reduces p-RPS6 in bladder cancer cells [118]
FRI00705	✓ a specific inhibitor of p70S6K1 (IC_50_ = 3 nM) and p70S6K2 (IC_50_ = 35 nM) and reduces p-RPS6 (S235/236) and p-RICTOR (T135) [254] ✓ reduces p-RPS6 (S235/236) and induces PD-L1 in H460 and HCC827 NSCLC cell lines [150]
FTI 277	✓ a farnesyl protein transferase (FPT) inhibitor [255] ✓ inhibits E_2_-induced p-RPS6 (S235/236) by blocking RHEB farnesylation [208]
FMK	✓ Pyrrolopyrimidine FMK (fluoromethylketone), an irreversible RSK inhibitor [256] ✓ inhibits serum-induced p-RPS6 (S235/236) [48]
Forskolin (colforsin)	✓ an activator of adenylyl cyclase (ADC) [257] ✓ induces p-RPS6 (S235/236) in a rapamycin-sensitive manner [82]
GNE-140	✓ a selective inhibitor of L-lactate dehydrogenase A chain (LDHA) (IC_50_ = 3 nM) and LDHB (IC_50_ = 5 nM) [258] ✓ reduces p-RPS6 (235/236) and p-p70S6K (T389) only in glycolysis-dependent cancer cell lines [114]
GSK28030371	✓ an allosteric inhibitor of PPM1D/WIP1 (IC_50 pp_= 6 nM) [259] ✓ reduces wire injury-induced p-RPS6 (S235/236) through the AMPK/mTORC1 pathway in mouse carotid arteries [92] ✓ reduces the PDGF-BB-induced p-RPS6 (S235/236) by inactivating mTORC1 through dephosphorylation of p-AMPKα (T172) in vascular smooth muscle cells [92]
Imatinib (STI571, CGP057148B, Gleevec)	✓ an inhibitor of PDGFR (IC_50_ = 100 nM), KIT (IC_50_ = 100 nM), and ABL1 (IC_50_ = 600 nM) [260] ✓ reduces BCR-ABL-mediated p-RPS6 (S235/236 and S240/244) via p70S6K inactivation in CML cells [86,102,103,104] ✓ upregulates PDCD4 in BCR-ABL CML cells but downregulates p-RPS6 (S235/236) and p-p70S6K (T389) [103] ✓ reduces p-RPS6 (S235/236) in the imatinib-sensitive CML cells but not in the imatinib-resistant cells [109]
IR (ionizing radiation)	✓ reduces p-RPS6 (S235/236) in NHDFs [100]
KN93	✓ a reversible, competitive inhibitor of CAMK2 [261] ✓ reduces p-RPS6 (S235/236 and S240/244) in leukemia cells [104]
KU-0063794	✓ a catalytic mTOR inhibitor (IC_50_ = ~10 nM for both mTORC1 and mTORC2) [262] ✓ reduces p-RPS6 (S240/244) [263]
LiCl (lithium chloride)	✓ an inhibitor of GSK3β [264,265] ✓ induces p-RPS6 (S240/244) [96]
Linsitinib (OSI-906)	✓ an inhibitor of IGF1R (IC_50_ = 35 nM), INSR (IC_50_ = 75 nM), and insulin receptor-related protein (IRR) (IC_50_ = 75 nM) [266] ✓ induces p-RPS6 in CRC cells through activation of p70S6K1, which is inactivated by PDCD4 [188]
LGB321	✓ a pan-PIM2 inhibitor with K_i_ of 1.0 pM for PIM1, 2.1 pM for PIM2, and 0.8 pM for PIM3 [267] ✓ reduces p-RPS6 (S235/236) in multiple myeloma cells [132]
Lonafarnib (SCH66336)	✓ an FPT inhibitor [268] ✓ reduces p-RPS6 (S235/236) through inhibiting RHEB farnesylation [269]
luteolin	✓ inhibits RSK1/2, leading to a reduction in p-RPS6 (S236) in SUM149PT cells [270]
LY294002	✓ the first small molecule identified to inhibit PI3Kα (IC_50_ = 0.5 μM), PI3Kδ (IC_50_ = 0.57 μM), and PI3Kβ (IC_50_ = 0.97 μM) and also inhibits CK2 (IC_50_ = 98 nM) and DNA-PK (IC_50_ = 1.4 μM) [271,272,273] ✓ reduces the NGF-induced p-RPS6 (S235/236) in rat PC12 cells [90]
Mesalamine (5-aminosalicylic acid, 5-ASA)	✓ an inhibitor of interleukin 1 (IL1)-stimulated RelA phosphorylation [274] ✓ inhibits PLD activity [212] ✓ a ligand for PPARγ [275] ✓ reduces p-RPS6 by inhibiting mTOR kinase activity in a PLD-dependent but not a PPARγ-dependent manner in CRC cells [212]
Metformin	✓ an activator of AMPK [276] ✓ reduces p-RPS6 (S235/236) in CRC cancer stem cells (CSCs) [214]
MK-2206	✓ an allosteric inhibitor of AKT1 (IC_50_ = 5 nM), AKT2 (IC_50_ = 12 nM), and AKT3 (IC_50_ = 65 nM) [277] ✓ reduces p-RPS6 (S235/236) and p-AKT (S473), and induces PD-L1 in an NSCLC cell line HCC827 [150]
ML281	✓ a selective inhibitor of STK33 (IC_50_ = 0.014 μM) [278] ✓ reduces p-RPS6 (S235) in a human SCLC cell line NCI-H446 both in vitro [143]
Nilotinib (AMN-107)	✓ an inhibitor of BCR-ABL (IC_50_ < 30 nM) [279] ✓ reduces p-RPS6 (S235/236 and S240/244) via the mTOR pathway [103] ✓ upregulates PDCD4 in BCR-ABL CML cells, while suppressing p-RPS6 (S235/236) and p-p70S6K (T389) [103]
Okadaic acid	✓ an inhibitor of the serine/threonine phosphatase PP2A (IC_50_ = 0.2 nM) and PP1 (IC_50_ = 19 nM) [280] ✓ induces p-RPS6 (S240/244) through the MAP4K3/mTORC1/S6K1 pathways by inhibiting PP2A that inhibits MAP4K3 in response to the amino acids’ withdrawal [117]
Omipalisib (GSK2126458)	✓ an inhibitor of PI3Kα (K_i_ = 0.019 nM), PI3Kδ (K_i_ = 0.024 nM), PI3Kγ (K_i_ = 0.06 nM), PI3Kβ (K_i_ = 0.13 nM), mTORC1 (K_i_ = 0.18 nM), and mTORC2 (K_i_ = 0.3 nM) [281] ✓ reduces p-RPS6 in melanoma cells with acquired resistance to dabrafenib in combination with dabrafenib and trametinib (GSK1120212) [243]
Oxamate	✓ an LDHA inhibitor that induces the G_2_/M cell-cycle arrest via reducing the CDK1/cyclin B1 pathway and promotes apoptosis by enhancing mitochondrial ROS generation in cancer cells [282,283] ✓ reduces p-RPS6 (235/236) and p-p70S6K (T389) only in glycolysis-dependent cancer cell lines [114]
L-norvaline	✓ an arginase inhibitor [284] ✓ inhibits TNFα-induced S6K1/RPS6 activation [94]
PD184352 (CI-1040)	✓ an MEK1/2 inhibitor with IC_50_ = 17 nM [285] ✓ inhibits p-RPS6 in the p21-induced senescent HT-p21 cells [286]
Phenformin	✓ an AMPK activator [287] ✓ reduces p-RPS6 (S235/236) (S240/244) in CRC cells [223]
PI-103	✓ an inhibitor for PI3K p110α (IC_50_ = 2 nM), p110β (IC_50_ = 3 nM), p110δ (IC_50_ = 3 nM), p110γ (IC_50_ = 15 nM), DNA-PK (IC_50_ = 23 nM), and mTOR (IC_50_ = 30 nM) [288] ✓ reduces p-RPS6 (S235/236, S240/244) and p-AKT (S473) in an SCLC cell line, H69 [131] ✓ reduces p-RPS6 (S235/236), p-mTOR (S2448), p-AKT (S473), p-GSK3β (S9), and p-BAD (S112) in TNBC cells [289]
PIK-75	✓ a specific inhibitor for DNA-PK (IC_50_ = 2 nM), PI3K p110α (IC_50_ = 5.8 nM), and PI3K p110γ (IC_50_ = 76 nM) [290,291] ✓ reduces p-RPS6 (S235/236 and S240/244) and p-AKT (S473) in the SCLC cell line H69 and concurrently reduces the cell viability by inducing apoptosis and autophagy [131]
PF-4708671	✓ an inhibitor of S6K1 (IC_50_ = 160 nM) [292] ✓ partially inhibits p-RPS6 in the p21-induced senescent HT-p21 cells [286] ✓ reduces p-RPS6 (S235/236) in imatinib-resistant CML cells [109] ✓ reduces p-RPS6 in prostate cancer cell lines PC3 and DU145 with a decrease in cell number and migration only in PC3 cells [293] ✓ reduces p-RPS6 (S235/236) and induces PD-L1 in H460 and HCC827 NSCLC cell lines [150]
PKC412	✓ a PKC inhibitor [294] ✓ reduces p-RPS6 (S235/236 and S240/244) in leukemia cells [104]
PMA (phorbol 12-myristate 13-acetate)	✓ a PKC activator [295] ✓ induces p-RPS6 (S235/236, S240/244) in a MEK-dependent mechanism [48] ✓ induces p-RPS6 (S236/236) via the PKCη/S6K axis in response to PMA, leading to rapamycin-insensitive cell proliferation [296]
Propranolol	✓ a competitive non-selective inhibitor of beta-adrenergic receptors (IC_50_ = 12 nM) [297] ✓ inhibits LPIN activity [179,298,299] ✓ inhibits p-AKT (S473) and p-RPS6 (S235/236) and induces accumulation of LC3-II and p62 in the prostate cancer cell line PC3, and sensitizes PC3 and HS578T to rapamycin [183]
RAME (rosmarinic acid methyl ester)	✓ an S6K1 inhibitor identified by a combined structure- and ligand-based virtual screening from a natural product library [237] ✓ reduces p-RPS6 (S235/236) in HeLa and SiHa cells and inhibits the interaction of S6K1 with mTOR and RPS6 [237] ✓ increases the cisplatin sensitivity of HeLa cells [237]
Rapamycin	✓ an allosteric mTOR inhibitor (IC_50_ = ~0.1 nM) [300] ✓ inhibits the serum-induced increase in p-RPS6 and translation of TOP mRNAs in Swiss mouse 3T3 cells [93] ✓ inhibits p-RPS6 (S235/236, S240/244) via S6K1 [48,301] ✓ inhibits p-RPS6 in p21-induced senescent HT-p21 cells [286] ✓ inhibits human CG- and forskolin-induced p-RPS6 (S235/236) [82] ✓ reduces p-RPS6 levels that induced by PD-L1 Ig [149] ✓ upregulates PDCD4 in BCR-ABL CML cells but downregulates p-RPS6 (S235/236) and p-p70S6K (T389) [103]
Resveratrol	✓ an inhibitor of quinone reductase 2 (IC_50_ = 88 nM) [302] ✓ an activator of SIRT1/2 [303] ✓ reduces p-RPS6 by direct inhibition of S6K, leading to suppression of autophagy [304] ✓ reduces TNFα-induced p-RPS6 (S235/236) in NIH/3T3 fibroblast cells, ameliorating inflammation [305] ✓ reduces doxorubicin-induced p-RPS6 (S240) via inhibition of S6K1 in neonatal rat cardiomyocyte, leading to suppression of doxorubicin-induced autophagy and cell death [252]
Sapanisertib (MLN0128, INK 128, TAK-228)	✓ a selective mTOR inhibitor (Ki = 1.4 nM) [251] ✓ reduces p-RPS6 (S235/236) and p-AKT (S473), and induces PD-L1 in cancer cells [150]
SB415286	✓ an inhibitor of GSK3α/β (IC_50_ = ~78 nM) [306] ✓ reduces p-RPS6 through inactivating S6K1 in breast cancer cells, colon cancer, kidney cells, osteosarcoma cells, and prostate cancer cells [83]
Selumetinib (AZD6244)	✓ an MEK1 inhibitor (IC_50_ = 14 nM) [307] ✓ reduces p-RPS6 (S235/235) in vemurafenib-sensitive melanoma cells, but not in resistance melanoma cells [308] ✓ synergistically reduces p-RPS6 (S235/235) with *RICTOR*-KD or *S6K1/2*-KD in vemurafenib-resistance melanoma cells [308]
SGI-1776	✓ an inhibitor of PIM1 (IC_50_ = 7 nM), FLT3 (IC_50_ = 44 nM), PIM3 (IC_50_ = 69 nM), and PIM2 (IC_50_ = 363 nM) [309] ✓ reduces p-RPS6 (S235/236) and p-70S6K (T389) in GBM LN229 cells [133] ✓ synergistically reduces p-RPS6 (S235/236) with alpelisib (BYL-719) in LN229 and U87 GBM cells and 83Me GBM mesenchymal stem cells, leading to synergistic inhibition of cell viability [133]
Silmitasertib (CX-4945)	✓ an inhibitor of CK2 (IC_50_ = 1 nM) [310] ✓ further reduces p-RPS6 (S235/236) in combination with knockdown of *CSNK2A1* (the gene encoding CK2α) or *CSNK2B* (the gene encoding CK2β) in a normal renal tubular epithelial HK-2 cells [110]
SRT2183	✓ a SIRT1 activator [311] ✓ reduces p-RPS6 (S240/244), p-AKT (S473), p-mTOR (S2448), and p-p70S6K (T389) in ovarian cancer OVCAR-3 and A2780 cells
Topotecan	✓ reduces p-RPS6 (S235/236) in various cancer cells in an AKT-dependent but p53- and AMPK-independent manner [100] ✓ reduces p-RPS6 (S235/236) in MEFs in a DNA-PK dependent manner [100]
Torin 1	✓ an inhibitor of mTORC1 (IC_50_ = 2 nM), DNA-PK (IC_50_ = 6.34 nM), and mTORC2 (IC_50_ = 10 nM) [312,313] ✓ reduces p-RPS6 (S235/236) and p-AKT (S473), and induces PD-L1 in an NSCLC cell line, HCC827 [150]
Torkinib (PP242)	✓ an mTOR inhibitor (IC = 8 nM) [314] ✓ reduces p-RPS6 levels that induced by PD-L1 Ig [149]
Trametinib (GSK1120212)	✓ an MEK1/2 inhibitor with an IC_50_ value of 0.92 and 1.8 nM, respectively [315] ✓ reduces p-RPS6 in naïve melanoma cells as a single agent treatment and dabrafenib resistant melanoma cells in combination with dabrafenib and trametinib (GSK1120212) [243]
U0126	✓ a MEK1/2 inhibitor with IC_50_ of 0.07 and 0.06 μM, respectively [316] ✓ inhibits p-RPS6 (S235/236) [48] ✓ inhibits p-RPS6 in the p21-induced senescent HT-p21 cells [286]
Vemurafenib (PLX4032)	✓ a BRAF^V600E^ inhibitor (IC_50_ = 31 nM); also inhibits Src-related kinase lacking C-terminal regulatory tyrosine and N-terminal myristylation sites (SRMS), activates CDC42 kinase (ACK), CRAF, MAP4K5, and Gardner-Rasheed feline sarcoma viral oncogene homology (FGR) with an IC_50_ value of 18, 19, 48, 51, and 63, respectively [317] ✓ reduces p-RPS6 (S235/235) in susceptible melanoma cells but not in resistance melanoma cells [308] ✓ synergistically reduces p-RPS6 (S235/235) with *RICTOR*-KD or *S6K1/2*-KD in vemurafenib-resistance melanoma cells [308]
Wedelolactone	✓ an inhibitor of IKK, leading to inhibition of NF-κB-mediated transcription [318] ✓ reduces p-RPS6 (S235/236) through decreasing H_2_O_2_-mediated p85S6K1 activity in a P3K/mTOR-independent manner [115]
WYE-354	✓ a catalytic mTOR inhibitor (IC_50_ = 5 nM) [319] ✓ reduces p-RPS6 (S240/244) [263]
YM-024	✓ a selective inhibitor for PI3K p110α (IC_50_ = 0.3 μM) [320] ✓ reduces p-RPS6 (S235/236 and S240/244) and p-AKT (S473) in the SCLC cell line H69, and concomitantly reduces the cell viability by inducing apoptosis and autophagy [131]

### 2.3. Subcellular Localization of RPS6

As a component of the 40S ribosome, RPS6 is transported from the cytoplasm, where its translation occurs, to the nucleolus, where it is assembled into the 40S ribosome [321]. Then, the 40S ribosome is released to the nucleoplasm before its transport into the cytoplasm through the nuclear pores. RPS6 has three putative NLSs (Figure 1), and removal of all these NLSs results in the failure of its nuclear import [322]. The functions of RPS6 in all these cellular locations remain largely unknown. 

In addition, p-RPS6 has been found in both the cytoplasm and nucleus [69], but differential nuclear/cytoplasmic distribution of p-RPS6, which depends on the phosphorylated sites, has also been demonstrated [323] with still unknown physiological significance. The nucleocytoplasmic localization of total (t)-RPS6 and p-RPS6 (S240/244) has been investigated. In *TSC1* wildtype MEFs, the level of p-RPS6 (S240/244) was regulated in a cell cycle-dependent manner; the lowest levels were observed in the G_1_ phase, a strong increase and a moderate decline in S phase, and the second peak in the G_2_/M phase [324]. The levels of p-RPS6 and the accompanying t-RPS6 were increased in the nucleus during the G_1_ phase. The nuclear level of p-RPS6 reached the maximum in the early S phase, whereas the level of t-RPS6 rapidly declined. Concomitantly, the cytoplasmic RPS6 was strongly upregulated and peaked in the mid (t-RPS6)-to-late S (p-RPS6) phase.

### 2.4. Proteasome-Dependent Degradation of RPS6

RPs have been found excessively expressed beyond the amount required for efficient ribosome biogenesis, and their levels are controlled through continuous proteasomal degradation in the nucleoplasm [325]. However, the corresponding E3 ligase responsible for RPS6 stability has not been identified yet. Although the *Drosophila* Pallbearer (PALL) has been identified as an F-box protein that regulates proteasome-dependent RPS6 turnover [326], the role of the human homolog has not been investigated yet (see Section 3.2.11. Functions of RPS6 in Other Higher Eukaryotes).

Cyclic accumulation of RPS6 in the nucleolus, in accordance with the cell cycle, has been reported; it starts in the S phase, culminates in the G_2_ phase, and diminishes in the M phase with the disintegration of the nucleoli [327]. In addition, mammalian RPS6 is dephosphorylated upon heat shock [328,329]. Heat shock protein 90 (HSP90) has been reported to interact with RPS3 and RPS6 and protect them from proteasomal degradation [330]. Consistently, the HSP90 inhibitor geldanamycin blocked the HSP90-RPS6 interaction and induced the degradation of RPS6. However, the mechanism for the regulation of RPS6 stability during cell cycle progress and by phosphorylation remains elusive.

Downregulation of t-RPS6 has been found in TNBC cells treated with the EGFR inhibitor (EGFRi) gefitinib and the MET inhibitor (METi) SU11274 [21] (see Section 5.2.1. RPS6-KD in Breast Cancer Cells). Although the reduction in t-RPS6 level was augmented until 16 h post-treatment, 4 h treatment of the cells with the proteasome inhibitor MG132 was not sufficient to inhibit the reduction in t-RPS6 level in the presence of gefitinib and SU11274 [21]. Further studies will be necessary to reveal the role of RPS6 stability in the combinatorial PKI therapies against cancer. 

## 3. Functions of RPS6

### 3.1. Ribosome Biogenesis and Protein Synthesis

RPS6 plays a role in the maturation of pre-rRNA. The siRNA-based *RPS6*-KD in HeLa cells resulted in the accumulation of 30S pre-rRNAs and the decrease in mature 18S rRNAs without perturbing the formation of mature 28S and 5.8S rRNAs [331]. Similar to this observation, in a mouse model with conditional deletion of both *Rps6* alleles in liver, the decease in 18S rRNAs was observed, and 34S pre-rRNAs (equivalents of human 30S pre-rRNAs) were accumulated in liver cells [332]. However, the hepatic hypertrophy but not hyperplasia in fasted animals in response to nutrients was not blocked by the absence of RPS6. A recent study has demonstrated that p-RPS6 is involved in the endonuclease processing of 30S pre-rRNAs in the nucleolus [327]. The splicing of 30S pre-rRNAs was only mediated by fully phosphorylated RPS6 in the C-terminal five serine residues. Additional genetic manipulations of *RPS6*, including tissue-specific knockouts of both alleles or conditional deletion of one allele, further support its indispensable role in the ribosome in the thymus, spleen, and lymph nodes [333]. More importantly, *Rps6* gene haploinsufficiency leads to embryonic lethality during gastrulation, preceded by failure in entering mitosis and induced apoptosis [334]. As in heterozygous *Rps6* T cells [333], the embryonic lethality of heterozygous *Rps6* mice might be due to a p53-mediated checkpoint during gastrulation [334].

P-RPS6 controls translation at the level of mRNA-binding in dividing cells [335,336,337]. P-RPS6 (S235/236) is also induced by RAS/RAF/ERK/RSK signaling, and p-RPS6 is recruited to the 7-methylguanosine cap structure [48].

RPS6 has been reported to play a role in the translation of mRNAs with a polypyrimidine tract at their 5′-terminal oligopyrimidine track (5′-TOP mRNAs) [338]. High levels of RPS6 have been found in primary diffuse large B-cell lymphoma (DLBCL) cell lines and patient samples. *RPS6*-KD reduced the proliferation of DLBCL cell lines [338] and increased the 5′-TOP mRNA translation [338,339]. A study reports that the translation of 5′-TOP mRNAs is downregulated by rapalog treatment, suggesting a role of p-RPS6 in the regulation of 5′-TOP mRNA translation [93]. Another study has reported that MEFs with the non-phosphorylated mutant RPS6 in all five serine sites (rpS6^P−/−^) and 70 kDa S6K1-null (p70S6K1^−/−^) display efficient translation of 5′-TOP mRNAs in response to mitogens [90]. RPS6 has been found to associate with 5′-TOP mRNAs, such as RPS8, RPL11, RPL16, and RPS24, through the 5′-TOP sequences to inhibit their translation [338]. *RPS6*-KD in the human breast cancer cell MCF7 and the human cervical carcinoma cell HeLa increases the number of these 5′-TOP mRNAs in actively translating polysomes.

### 3.2. Extra-Ribosomal Functions of RPS6

**Figure 3:**
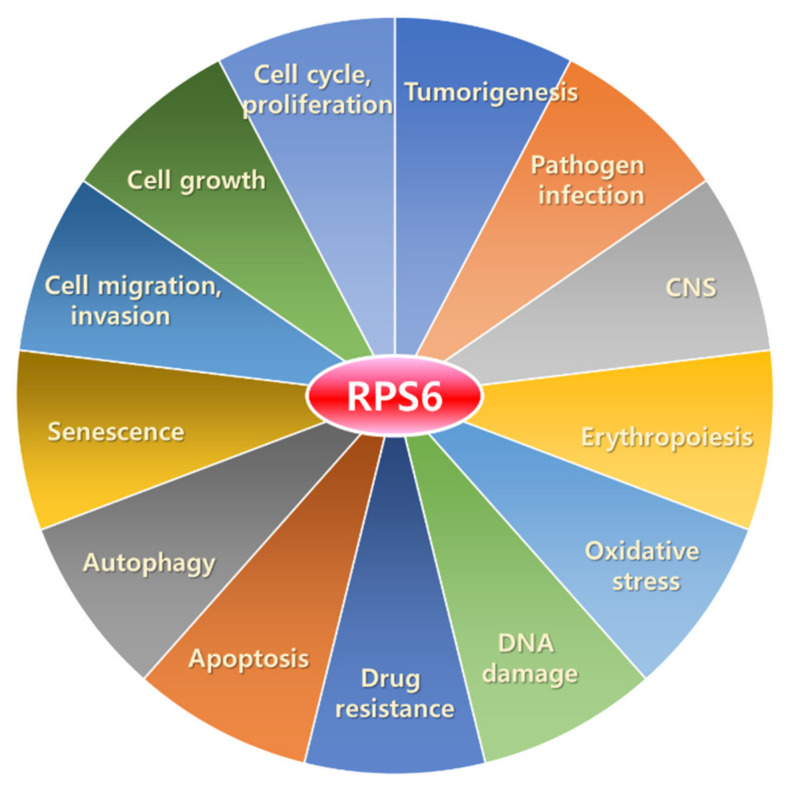
Extra-ribosomal functions of RPS6.

In addition to the roles in translational control, RPS6 has extra-ribosomal functions (Figure 3). A study has reported that approximately 5% of endogenous RPS6 is detected in ribosome-free subcellular fractions [340]. Here, we briefly describe the functions of RPS6 in various cellular processes.

#### 3.2.1. Regulation of Cell Cycle, Proliferation, and Growth

The relationship between p-RPS6 and cell cycle regulation was first identified in *Xenopus* eggs [341]. Hyperphosphorylation of RPS6 was correlated with the oncogenic RAS-induced cell-cycle arrest.

RPS6 deficiency has been demonstrated to induce the p53-dependent cell-cycle arrest that is antagonized by the depletion of p53 [339]. This induction of p53 is dependent on the upregulation of ribosomal protein L11 (RPL11). Loss of RPS6 disrupts the 40S ribosome biogenesis, leading to selective upregulation of the translation of 5′-TOP mRNAs [339]. RPL11, which is translated from one of these 5′-TOP mRNAs, binds to MDM2, the E3 ligase of p53. Under normal conditions, the activity of the tumor suppressor p53 is tightly regulated by MDM2, through a ubiquitin-dependent proteasome pathway [342,343]. RPL11-MDM2 association stabilizes and activates p53 by preventing MDM2-p53 binding [6]. Induction of p53 led to the expression of its target genes including cyclin-dependent kinase inhibitor 1a (*Cdkn1a*; the gene encoding p21), BCL2-associated X (*Bax*), and *Mdm2*, in the liver of *Rps6*-deleted mice [339]. Previous results also support the p53-dependent cell-cycle arrest and apoptosis in other tissues with the deletion of one *Rps6* allele [333,334]. Notably, *RPS6*-KD is also known to upregulate RPL11 in the human breast cancer cell line, MCF7, and the human cervical carcinoma cell line, HeLa [338].

Failure of hepatic cell proliferation or cyclin E induction after partial hepatectomy has been reported in RPS6-deficient mice despite the presence of active cyclin D-CDK4 complexes [332]. These results suggest the induction of a checkpoint control by abrogation of 40S ribosome biogenesis, leading to the prevention of cell cycle progression. The depletion of RPS6 induced the p53-dependent cell-cycle arrest [339]. Newborn rpS6^P−/−^ mice have been demonstrated to contain high DNA content [77]. In addition, rpS6^P−/−^ MEFs displayed a shorter population-doubling time with a short G_1_ phase compared with those of the wild-type MEFs. However, the mechanism of its regulation of cell proliferation remains to be determined.

The suppressive effect of short hairpin RNA (shRNA)-based *RPS6*-KD on cell proliferation has been observed to increase with time in two lung cancer cell lines, A549 and H520 [37]. Inversely, the expression of senescence-associated β-galactosidase (SA-β-gal) was increased by *RPS6*-KD in both cell lines. Additionally, *RPS6*-KD increased the number of cells in the G_0_/G_1_ phase and decreased the number of cells in the G_2_/M phase, concurrently with reduced levels of p-RB and cyclin D1, whereas no significant changes were observed in the levels of cyclin A, cyclin E, and total RB. More interestingly, the levels of CDK inhibitors (CKIs), including p16, p21, p27, and p57, were increased. However, *RPS6*-KD did not induce apoptosis and altered the expression of B-cell lymphoma-extra large (BCL-xL), BAX, and caspase-3. The anticancer effect of *RPS6*-KD was reproduced in a xenograft model: A549 lung cancer cells with *RPS6*-KD resulted in reduced tumorigenicity with an increase in the number of SA-β-gal(+) cells in xenograft tissues. Similar changes were observed in the expression of cell cycle regulators and CKIs [37].

Functional implications of RPS6 in cell cycle regulation were also demonstrated by *RPS6*-KD in a variety of cancer cells. *RPS6*-KD induced G_0_/G_1_ cell cycle-arrest in human NSCLC cells [37,38], ovarian cancer cells [40], and drug-resistant melanoma cells [39] (see Section 5.2. RPS6-KD in Cancer Cells).

The levels of p-RB, cyclin D1, cyclin E, CDK2, CDK4, or CDK6 by *RPS6*-KD were reduced in NSCLC cells [38], ovarian cancer cells [40], and drug-resistant melanoma cells [39]. Hypophosphorylated RB inhibits cell cycle progression by binding to the transcription factor E2F and repressing the transcription of E2F target genes that are required for G_1_-to-S transition [36]. The phosphorylation of RB is mediated by the cyclin D/CDK4/6 complex and derepresses the transcription of E2F target genes [36]. It remains to be studied how RPS6 regulates the G_0_/G_1_ checkpoint regulators in NSCLC, ovarian cancer cells, and melanoma cells. In contrast, the levels of the CKIs p21 and p27 were increased by *RPS6*-KD in NSCLC cells [38]. These CKIs inhibit CDK activity either by disrupting the CDK4/6-cyclin complexes (p16) or by binding to both the cyclin and CDK in the complexes (p21, p27, and p57) [344]. Inversely, overexpression of RPS6 in the normal human bronchial epithelial (HBE) cell line promoted the cell proliferation with concurrent increases in the levels of p-RPS6, p-RB, cyclin D1, cyclin E, CDK2, and CDK4 and decreases in CKIs, p21 and p27, and the number of cells at the G_0_/G_1_ phase [38].

It has been demonstrated that cell growth (increase in size/volume) is regulated mainly by the mTORC1 pathway. RPS6 phosphorylation by S6K1 has been demonstrated to be directly involved in the positive control of cell size. Various cells derived from rpS6^P−/−^ mice, including MEFs, fetal liver cells, pancreatic β-cells [345], and muscle myotubes [78], have been reported to be significantly smaller than the wild-type controls. However, pancreatic acinar cells displayed a similar size regardless of S6K1 deficiency [346] or RPS6 phosphorylation defect [77].

#### 3.2.2. Regulation of Cell Migration

The migration of NSCLC cells is also reduced by *RPS6*-KD, which has been shown to downregulate the level of proteins involved in cell migration, including N-cadherin, vimentin, matric metallopeptidase-9 (MMP-9), MMP-2, and p-paxillin. Conversely, E-cadherin is increased by *RPS6*-KD [38]. In contrast, the migration of HBE cells is enhanced by RPS6 overexpression. Additionally, RPS6 overexpression upregulates N-cadherin, vimentin, MMP-2, and p-paxillin in HBE cells [38]. Induction of the epithelial–mesenchymal transition (EMT) markers suggests that overexpression of RPS6 contributes to metastasis of cancers [347].

Cell migration is also linked to angiogenesis and the tumor microenvironement. In fact, activation of the mTOR/RPS6 pathway has been associated with cancer cell survival, inflammation, and neoangiogenesis through various upstream regulators [84,95,310,348]. Since tumor growth is a result of the constant crosstalk between a tumor and its surrounding microenvironment, leading to neoangiogenesis and immune escape, further studies on the role of RPS6 in this crosstalk contribute to developing additional strategies combining anti-angiogenic therapy and immunotherapy against cancer [349,350].

#### 3.2.3. Regulation of Apoptosis

Unphosphorylated RPS6 has been demonstrated to induce apoptosis via a mechanism involving the tumor necrosis factor-related apoptosis-inducing ligand (TRAIL) by inducing the expression of death receptor 4 (DR4) [340] (see Section 5.2.2. RPS6-KD in Cervical Carcinoma Cells and Section 5.2.7. RPS6-KD in Hematopoietic Cancer Cells). MEFs that express unphosphorylated RPS6 have been demonstrated to be more sensitive to TRAIL-induced apoptosis than the control MEFs. Similarly, the human HCC cell line, SK-HEP-1, expresses a low level of p-RPS6 and is more vulnerable to TRAIL-induced apoptosis than other tumor cells that express a high level of p-RPS6. Consistently, ectopic expression of the phospho-defective RPS6 mutant (RPS6^SS235/236AA^) in HeLa cells resulted in an increase in TRAIL-induced apoptosis compared to that of the phospho-mimic RPS6 mutant (RPS6^SS235/236DD^) [340]. Conversely, the overexpression of RPS6^SS235/236DD^ had no effect on the TRAIL-induced apoptosis. Since S6K1, the upstream kinase of RPS6, has anti-apoptotic activity, other apoptotic regulators, rather than RPS6, may contribute to the S6K1-dependent apoptosis induced by TRAIL [340]. More importantly, the N-terminus of RPS6 (aa 1–70) without the phosphorylation residues has been reported to carry pro-apoptotic activity to sensitize HeLa cells to TRAIL. These results imply a phosphorylation-dependent negative regulatory effect of the C-terminus of RPS6 on the pro-apoptotic activity of RPS6 [340].

DNA damage-regulated autophagy modulator protein 1 (DRAM1) is a direct target of p53 during DNA damage. It induces autophagy and is essential for the p53-dependent apoptosis [351]. Recently, negative regulation of p-RPS6 by DRAM1 has been reported [166]. Overexpression of DRAM1 downregulated the levels of p-RPS6 (S235/236) and p-RPS6 (S240/244) in an mTORC1-dependent manner in HEK293T cells. DRAM1 was also found to be localized at the plasma membrane to regulate IGF1R phosphorylation. Additionally, the overexpression of DRAM1 reduced the viability and inhibited the colony formation of the human colon cancer cell line SW480 [166]. The role of the DRAM1/RPS6 axis in apoptotic regulation remains to be determined.

#### 3.2.4. Regulation of Drug Resistance

The overexpression of RPS6 confers intrinsic or acquired drug resistance to cancer cells [39,352,353,354,355,356,357,358,359,360]. RPS6 has been reported to confer drug resistance through nuclear factor erythroid 2-related factor 2 (NRF2) [354]. NRF2 is a master transcription factor that activates genes during oxidative stress response, detoxification, and drug resistance [361,362,363,364,365,366,367,368]. In human epidermal growth factor receptor 2 (HER2)-amplified gastric cancer (GC) cell lines with resistance to the HER2 inhibitors (HER2is) lapatinib and trastuzumab, *RPS6*-KD reduced the cell viability and the cellular resistance to HER2is [354]. More importantly, *RPS6*-KD led to downregulation in the levels of mRNAs of NRF2 target genes such as aldo-keto reductase (AKR) family 1 member B10 (*AKR1B10*), C1 (*AKR1C1*), and C2 (*AKR1C2*). The RPS6-NRF link was further confirmed by pharmacological inhibition of p-RPS6 in a xenograft model; treatment of the xenografted animals with the PI3K/mTOR inhibitor GSK458 alongside lapatinib reduced the growth of the HER2i-resistant tumor concurrently with downregulation of p-RPS6 and NRF2 proteins and the *AKR1C1, AKR1C2,* and *AKR1B10* mRNAs in the xenograft tumor [354]. Human AKRs are NAD(P)H-dependent oxidoreductases, and their overexpression leads to drug resistance [369]. Previously, RPS6 has been reported to induce the translation of *NRF2* by binding to the *NRF2* mRNA through interaction with Sjögren syndrome antigen B (SSB) [370]. Notably, HER2 induced drug resistance in human breast cancer cells by direct physical binding to and activation of NRF2 [361].

#### 3.2.5. Roles in the DNA Damage Response

The expression of rpS6^P−/−^ in mice with the oncogenic Kristen rat sarcoma (*KRAS*) gene background has shown an increase in p53 expression along with the increased staining of phosphorylated H2A histone family member X (γ-H2AX) and p53-binding protein 1 (53BP1) in areas of acinar ductal metaplasia [17]. First, the administration of rapamycin, the selective inhibitor of mTORC1, once every other day for 1 month to mice implanted with a 7,12-dimethylbenz[a]anthracene (DMBA)-soaked cotton pledget in the pancreas significantly decreased the score of pancreatic intraepithelial neoplasia (PanIN) lesions compared with the score in the untreated mice. Histological examination has revealed that no RPS6 phosphorylation is observed in the pancreas of DMBA-treated mice when treated with rapamycin, whereas strong p-RPS6 is observed without rapamycin treatment. Transgenic mice expressing the oncogenic *KRAS*^G12D^ in the pancreatic epithelium develop PanIN lesions that infrequently progress to pancreatic ductal adenocarcinoma (PDAC) [371]. These mice have a strong and uniform expression of p-RPS6 in the acinar cells [17]. Additionally, rpS6^P−/−^ mice treated with DMBA showed attenuated development of PanIN lesions. Furthermore, mice with both a *KRAS*^G12D^ mutation and rpS6^P−/−^ had a significantly lower score of PanIN lesions compared with the score in the mice with a *KRAS*^G12D^ mutation and one or two wild-type *RPS*6 alleles. Further analysis revealed that frequent p53-positive cells were detected in mice with both *KRAS*^G12D^ mutation and rpS6^P−/−^. Finally, the DNA damage markers, γ-H2AX and 53BP1, were highly expressed in the mice with both *KRAS*^G12D^ mutation and rpS6^P−/−^. γ-H2AX is recruited to the chromatin domains near DNA double-strand breaks (DSBs) [372] and 53BP1 is colocalized with γ-H2AX and required for p53 accumulation in response to DNA damage [373]. Taken together, these results suggest the potential role of p-RPS6 in the attenuation of DNA damage in a mutant *KRAS* background, leading to the reduction of p53-dependent tumor suppression [17]. In relation to this observation, BEZ235, a dual inhibitor of PI3K and mTOR, has previously been reported to downregulate p-53BP1 (S25) and 53BP1 foci induced by the poly (ADP-ribose) polymerase inhibitor (PARPi) olaparib in breast cancer type 1 susceptibility protein (*BRCA1*)-mutant breast cancer cells [374].

ATM, the master regulator of the DNA damage response, is a serine/threonine protein kinase [375]. Upon DSB formation, ATM is phosphorylated and activated to induce cell-cycle arrest, DNA repair, or apoptosis. The S247 of RPS6 is a substrate of ATM in response to UV irradiation or the treatment with the genotoxic drug doxorubicin and is inhibited by the ATM inhibitor KU-55933 [57]. ATM-mediated RPS6 phosphorylation has been reported to be S6K-independent.

Phosphorylation of RPS6 has been reported to attenuate DSBs in *BRCA1*-deficient breast cancer cells both in vitro and in vivo [376]. In olaparib-resistant *BRCA1*-deficient breast cancer cells, ionizing radiation (IR) decreased the number of γ-H2AX foci and increased the number of DNA repair protein RAD51 homolog 1 (RAD51) foci compared with the numbers of them in the parental cells. A remarkable increase in p-RPS6 is the distinct feature of olaparib-resistant *BRCA1*-deficient cells, and abrogation of RPS6 phosphorylation completely reverses the formation of foci for γ-H2AX or RAD51. These results suggest that p-RPS6 is crucial to load the RAD51 recombinase onto DNA damage sites in *BRCA1*-deficient cells. In fact, BRCA1 is indispensable for RAD51 loading onto DNA damage sites, leading to homologous recombination repair (HRR) [377]. More importantly, data support that p-RPS6 confers olaparib-resistance to *BRCA1*-deficient breast cancer cells [see Section 4.3.4. RPS6 in Resistance to PARP Inhibitors (PARPis)].

#### 3.2.6. Response to Oxidative Stress

Insulin has been demonstrated to induce the interaction of mTORC2 with the ribosome, leading to enhancement of mTORC2 activity in a translation-independent manner [378]. It has been reported that RPS6 plays a cardioprotective role in response to oxidative stress [52]. IPC, and insulin or opioid treatment, induced phosphorylation of RPS6 at S235/236 through the AKT/mTORC1/S6K pathway in perfused mouse hearts or neonatal rat ventricular myocytes. The p-RPS6 interacted with the rapamycin-insensitive companion of mTOR (RICTOR) to enhance mTORC2 kinase activity. *RPS6*-KD reduced the insulin-induced phosphorylation of mTORC2 and AKT (S473). Inversely, RPS6 overexpression upregulated p-AKT (S473). Additionally, *RPS6*-KD abrogated insulin-induced cardioprotection against the H_2_O_2_-induced oxidative stress [52]. Interestingly, S6K1 negatively regulates mTORC2 via the phosphorylation of RICTOR in the mouse adipocyte cell line 3T3-L1 [379]. Taken together, mTORC2 activity may be tightly controlled by RPS6-mediated positive feedback and S6K-induced negative feedback.

#### 3.2.7. RPS6 in Cellular Senescence

Cellular senescence is one of the nine hallmarks for aging, which is characterized by constant G_1_ cell-cycle arrest and an inflammatory response, the senescence-associated secretory phenotype (SASP) [380,381,382]. The mTORC1/S6K pathway has been recognized as the key signaling pathway responsible for aging and cellular senescence [383,384]. Since RPS6 is the downstream effector of this mTORC1/S6K pathway, p-RPS6 has been used as a marker for aging and premature senescence [385].

Contrary to this notion, a study found that there are no significant differences in the levels of mTOR or p-mTOR (S2448) between young and old human dermal fibroblasts (HDFs), whereas downregulation of S6K1, p-S6K1 (T389), and p-RPS6 was observed in replicative senescent HDFs [386]. In addition, p-RPS6, S6K1, and p-S6K1 were also downregulated in HDFs with autophagy impairment-induced premature senescence (AIPS) that was induced by siRNA-based knockdown of the autophagy related 7 (*ATG7*) or lysosome-associated membrane glycoprotein 2 (*LAMP2*). Interestingly, reactive oxygen species (ROS) scavenging (through treatment of the antioxidant *N*-acetyl cysteine (NAC)) or p53 inhibition (either through treatment of a p53 inhibitor, pifithrin-α, or knockdown) restored the levels of p-RPS6, S6K1, and p-S6K1 in AIPS HDFs. However, the exact roles of RPS6 and its phosphorylation in cellular senescence have not been elucidated yet.

#### 3.2.8. Roles of RPS6 in Erythropoiesis

Congenital (Diamond-Blackfan anemia; DBA) and acquired (5q-syndrome) hypoproliferative macrocytic anemia share a common erythroid phenotype of RP haploinsufficiency [387]. Although mutations of the *RPS6* gene have not been reported in this anemia, mice lacking one *Rps6* allele postnatally display features of the 5q-syndrome, such as macrocytic anemia, erythroid hypoplasia, and megakaryocytic dysplasia with thrombocytosis [388]. Additionally, mice with heterozygously deleted *Rps6* have also been demonstrated to phenocopy the 5q-syndrome [389]. However, the clinical significance of these findings remains elusive.

#### 3.2.9. Roles of RPS6 in the Central Nervous System

Increasing evidence indicates p-RPS6 as a marker of neuronal activation during synaptic plasticity [390,391]. Various pharmacological stimuli also induced the p-RPS6 in neurons [392]. For example, massive phosphorylation of RPS6 at S235/236 and S240/244 by a proconvulsant drug, such as kainite, pilocarpine, pentylenetetrazol, or dopamine D1 receptor (DRD1) agonist SKF81297, has been demonstrated. In addition, drugs of abuse and antipsychotics also regulate RPS6 phosphorylation [392]. Interestingly, the p-RPS6 level in schizophrenia is reduced [393]. Elevated expression or phosphorylation of RPS6 has also been found in multiple sclerosis [394]. The roles of RPS6 in diseases of the central nervous system remain to be determined.

#### 3.2.10. Roles of RPS6 in Response to Infection

Mounting evidence suggests the roles of RPS6 during pathogen infection [395]. The induction of p-RPS6 in HeLa cells by vaccinia virus infection was reported as early as 1976 [396].

RPS6 has been demonstrated to be associated with latency-associated nuclear antigen (LANA). LANA is a multifunctional protein of Kaposi’s sarcoma-associated herpesvirus (KSHV) that is tightly associated with primary effusion lymphoma (PEL), Kaposi’s sarcoma I, and multicentric Castleman’s disease (MCD) [397]. RPS6 binds to the N-terminal domain of LANA in a nucleic acid-independent manner in a PEL cell line, BC-3 [398]. Interestingly, RPS6 increased the transcriptional activity of the LANA protein on the LANA promoter. Moreover, shRNA-based *RPS6*-KD reduced the stability of the LANA protein, while increasing the stability of p53 in BC-3 cells. The half-life of the LANA protein was markedly reduced from several days [399] to 0.6 h by *RPS6*-KD [398]. Consistent with the increased p53 stability, the upregulation of p21 protein levels was also induced by *RPS6*-KD. These effects led to anti-proliferative effects in BC-3 cells [399]. The underlying mechanism of the RPS6-dependent regulation of LANA stability is subject to further study.

The open reading frame 45 (ORF45) of KSHV has been reported to induce p-RPS6 (S235/236) by directly activating RSKs through the mTORC1/S6k-dependent signaling [400]. Phosphorylation of RPS6 and eukaryotic translation initiation factor 4B (eIF4B) through the ORF45/RSK axis has been suggested to contribute to the translational control during KSHV lytic replication.

An indispensable role of RPS6 has been found in hepatitis C virus (HCV) propagation. HCV is a single-stranded RNA virus whose translation is initiated in a cap-independent manner by its internal ribosome entry site (IRES) [401]. Reduction of the 40S ribosome abundance by *RPS6*-KD was reported to selectively suppress HCV IRES-mediated translation without affecting the translation of the host cells [402]. Only knockdown of the 40S *RPS* genes, but not the 60S *RPL* genes, of the ribosomal subunit inhibited HCV translation.

Protein phosphatase 2A (PP2A) is a heterotrimeric serine/threonine phosphatase, which is composed of a structural A subunit, a catalytic C subunit, and >20 regulatory B-type subunits resulting in >80 different PP2A holoenzymes [403]. The composition and function of the PP2A holoenzyme are regulated through methylation and demethylation by leucine carboxyl methyltransferase 1 (LCMT1) and protein phosphatase methylesterase 1 (PME1), respectively [404]. Cellular transformation either by polyomavirus or by simian virus 40 (SV40) occurs through activation of p-RPS6 (S235/236) via inhibiting PP2A by replacing the regulatory B-type subunits from the PP2A heterotrimer by viral oncoproteins such as the polyomavirus middle (PyMT) and small (PyST) tumor antigen and the SV40 small tumor antigen (SVST) [137]. PyST and SVST preferentially bind to PP2A instead of the methylated PP2A catalytic subunit, which is mediated by LCMT1, whereas the binding of PyMT is not. The methylated PP2A downregulates p-RPS6 by inactivating AKT and S6K. The methylated PP2A also reduced p-RPS6 in an S6K-independent manner. Similarly, PyMT and PyST enhance RPS6 phosphorylation [405,406].

RPS6 phosphorylation is also increased in cells infected by pathogens such as Rift Valley Fever (RVF) Virus, Herpesvirus, *Plasmodium*, and *Toxoplasma* [407,408,409,410]. Pharmacological inhibitions of the mTORC1/S6K/RPS6 pathway inhibits the infection or pathogenesis of these pathogens [407,408]. Additionally, p-RPS6 is activated by an unknown regulator of the non-canonical pathway in *Plasmodium*-infected hepatocytes [407]. These data suggest that parasites override the RPS6 pathway to support their growth in infected cells.

#### 3.2.11. Functions of RPS6 in Other Higher Eukaryotes

In the plant *Arabidopsis*, RPS6 negatively regulates rDNA transcription by associating with the histone deacetylase, AtHD2B [411], which might be antagonized by the interaction of RPS6 with the histone chaperone, AtNAP1 [412].

RPS6 has been identified as a negative regulator of efferocytosis in *Drosophila*. Efferocytosis is a process of clearing apoptotic cells via phagocytosis by professional or amateur phagocytes [413]. Since approximately 3 × 10^11^–5 × 10^11^ cells in our body die daily via apoptosis, clearing dead cells is crucial to maintain tissue homeostasis [414,415]. PALL is an F-box protein in *Drosophila*, and a component of the Skp-Cullin-F-box (SCF) E3 ubiquitin ligase complex [416]. PALL has been found to bind to p-RPS6 to promote RPS6 ubiquitylation and proteasomal degradation [326]. Degradation of RPS6 promoted efferocytosis through Rac family small GTPase 2 (RAC2) activation and F-actin remodeling in *Drosophila* cells. Consistently, *RPS6*-KD enhanced the efferocytosis and RPS6 overexpression reduced the clearance of apoptotic cells. Although F-box only protein 28 (FBXO28) has been identified as a human homolog of PALL [417], whether FBXO28 binds to and degrades p-RPS6 in mammalian cells remains to be determined.

## 4. RPS6 in Cancer

Although it still needs further investigation whether p-RPS6 is just a byproduct of tumorigenic pathway activation or a prerequisite for tumorigenesis in various types of cancers, we provide evidence here supporting the potential roles of RPS6 in tumorigenesis in humans and suggesting this protein as a potential therapeutic target against cancer.

### 4.1. Roles of RPS6 in Tumorigenesis

A knock-in mouse model of a non-phosphoylatable RPS6 mutant (rpS6^P−/−^) has demonstrated that p-RPS6 is indispensable to develop PDAC in *KRAS*^G12D^ mutation background [17]. The importance of p-RPS6 in tumorigenesis was further demonstrated in mice expressing constitutively active AKT (MyrAKT1) in pancreatic β-cells in the background of rpS6^P−/−^. Defects in the phosphorylation of RPS6 rescued the MyrAKT1-induced reduction in the nuclear level of p27 [418], a CKI [419,420]. In addition, p-RPS6 deficiency reduced the development of the MyrAKT1-induced hyperplasia and tumor formation in β cells (insulinoma). RPS6 phosphorylation deficiency also increased the overall protein synthesis with concomitant reduction in translation fidelity, leading to unexpected resistance to proteotoxic (MG132) or genotoxic (etoposide) stress-induced apoptosis in MyrAKT1-expressing cells. However, p-RPS6 deficiency failed to inhibit, and instead enhanced, the MyrAKT1-triggered aneuploidy, and increased the number of β cells and amount of insulin secretion [418]. Although several nuclear proteins, including PC4 and SFRS1-interacting protein 1 (PSIP1), serine/arginine-rich splicing factor 1 (SRSF1), and DNA topoisomerase IIβ (TOP2B), have been identified as binding partners of unphosphorylated RPS6, the functional significance of these interactions has not been elucidated yet [418]. Since rpS6^P−/−^ could not prevent thymic lymphomatogenesis in mice with a constitutively active AKT2 in immature T cells [421], the anti-tumorigenic effect of rpS6^P−/−^ may be tissue-specific.

P-RPS6 has been reported to promote the translation, not transcription, of hypoxia-inducible factor 1-alpha (HIF1α) upon the activation of an environmental carcinogen, arsenite, leading to carcinogen-induced transformation of the normal mouse epidermal Cl41 cells [422]. Arsenite treatment induced the increase in p-RPS6 (S235/236). P-RPS6-dependent HIF1α expression was negatively regulated by the CKI, p27, via the RAS/RAF/MEK/ERK/RSK pathway, but not via the AKT/S6K pathway. P27 is a cell cycle regulator that binds and inhibits cyclin D/CDK4 [423,424], cyclin D/CDK6 [425], and cyclin E/CDK2 complexes [426]. *CDKN1B* (the gene encoding p27)-KD induced p-RPS6 even in the absence of arsenite. Arsenite-induced p-RPS6 was augmented by *CDKN1B*-KD and in an arsenite dose-dependent manner. Mutational analyses further demonstrated that the induction of HIF1α translation in p27-deficient cells was p-RSP6 (S235/236)-dependent. Interestingly, *CDKN1B* depletion abrogated the expression of PHPLL, a negative regulator of RAS [427], RAF1 [428], and AKT [429,430], resulting in the activation of the RAS/RAF/MEK/ERK/RSK pathway [422]. Notably, SRC, the upstream regulator of the RAS/RAF/MEK/ERK pathway, phosphorylates p27 and induced its proteolysis [431]. In addition, hypoxia is an important inducer of neoangiogenesis, and activated RPS6 may play an important role in the crosstalk between endothelial cells and tumor cells in the tumor microenvironment [84,95,310,348]. For example, it has been reported that RPS6 is activated by tumor microenvironment conditions and hypoxia in endothelial cells [432].

### 4.2. RPS6 as a Predictive Marker in Cancers

**Figure 4:**
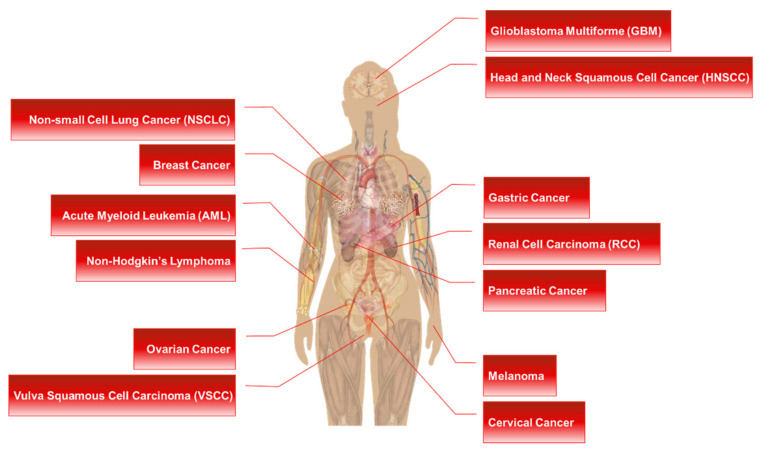
Cancers associated with high levels of RPS6 or p-RPS6. A variety of cancers has been reported to be associated with high levels of RPS6 or p-RPS6. The image of the human body adopted from https://www.pikpng.com/downpngs/iwmhwRb_female-chest-anatomy-diagram-female-human-anatomy-human/, accessed on 1 June 2021.

High-level phosphorylation and/or overexpression of RPS6 has been reported in various types of cancers, including acute myeloid leukemia (AML) [433], breast cancer [434], cervical cancer [435], esophageal squamous cell carcinoma (ESCC) [436], GC [437], glioblastoma multiforme (GBM) [438], HNSCC [439], melanoma [440], non-Hodgkin’s lymphoma [338], NSCLC [38], oral squamous cell carcinoma (OSCC) [441,442], ovarian epithelial cancer (OEC) [40,443], pancreatic cancers [13,17,444], renal cell carcinoma (RCC) [445], sarcoma [446], and vulva squamous cell carcinoma (VSCC) [447]. More interestingly, mTOR-independent phosphorylation of RPS6 was frequently identified in primary central nervous system lymphoma (PCNSL) and DLBCL [338,355]. PASK, but not RSK, was found as a potential kinase of RPS6 in these lymphomas [355]. Additionally, RPS6 has been proposed as a predictive biomarker in cancers. The level of RPS6 or p-RPS6 was also correlated with the pathological grade and/or disease progression in distinct human cancers (Figure 4). The change in the expression level and the status of p-RPS6 and/or t-RPS6 could be monitored by various methods to predict drug response and resistance, and disease progression after drug treatment [440].

#### 4.2.1. RPS6 in Acute Myeloid Leukemia (AML)

The shRNA-based knockdown of *CSNK1* (the gene encoding CK1) downregulated p-RPS6 (S244/247) concurrently with increased p53 activity in primary mouse MLL-AF9 leukemia cells [448]. *CSNK1*-KD had anti-leukemia efficacy both in vitro and in vivo. The anti-leukemic effects of *CSNK1*-KD were further supported by the rescue of CK1 functions by an shRNA-resistant *CSNK1* cDNA, which inhibits the anti-leukemic effects of *CSNK1*-KD, whereas kinase-dead *CK1^D136N^* [449] cDNA did not [448]. The regulation of RPS6 activity by CK1-mediated phosphorylation has been reported previously [56]. Interestingly, *CSNK1*-KD also induced the levels of p53 and p21 with concurrent induction of apoptosis and G_1_ arrest in MLL-AF9 leukemia cells [448]. Previously, it has been demonstrated that dephosphorylation of RPS6 causes p53 activation in pancreatic cancer cells in response to DNA damage [17]. In addition, RPS6 depletion has been reported to induce p53-dependent cell-cycle arrest [339]. The p53 induction was mediated by the upregulation of RPL11, which binds to and inhibits MDM2 in RPS6 depleted cells [6,339] (see Section 3.2.1. Regulation of Cell Cycle, Proliferation, and Growth). Overexpression of a phosphomimetic mutant RPS6^S5D^ [56] partially rescued the *CSNK1*-KD-induced proliferation defect in leukemia cells [448]. Taken together, the relationship between p-RPS6 and p53 in AML or other cancer cells will be an interesting topic in the future.

#### 4.2.2. RPS6 in Breast Cancer

High levels of p-RPS6 (S235/236), but not p-ERK (T202/Y204) or p53, have been found to strongly correlate with high Ki-67 expression in ER(+)/HER2(−) breast cancer clinical samples [434]. However, there was no significant difference in relapse-free survival (RFS) between cancer patients with high p-RPS6 levels and those with low p-RPS6 levels. In addition, high-level expression of p-RPS6 (S240) correlated with high Ki67 expression and short overall survival (OS) of breast cancer patients [450].

The t-RPS6 protein has been reported to be downregulated in TNBC cells treated by the EGFRi gefitinib and the AKT inhibitor MK2206 [451]. TNBC cells, especially mesenchymal stem-like (MSL) subtype cells, have intrinsic resistance to EGFRi [21,451,452,453,454,455,456]. Treatment of gefitinib alone did not reduce the viability or colony-forming ability of the MSL subtype cells [21,451,455]. The combination of gefitinib and MK2206 induced synergistic anti-proliferative and anti-colony forming effects in HS578T and MDA-MB-231 cells. No significant suppression of the PI3K/AKT and MAPK pathways was observed in TNBC cells treated with gefitinib alone. The treatment of MK2206 alone reduced the levels of p-AKT (S473), p-mTOR (S2448), p-PRAS40 (T246), p-4E-BP1 (T37/46), and p-RPS6 (S235/236). Interestingly, MK2206 alone induced downregulation of t-RPS6 [451]. The reduction of t-RPS6 was induced by the gefitinib+MK2206 treatment in a dose-dependent manner. Since no significant change in the level of p-ERK1/2 (T202/Y204) was observed, RSK may not contribute to the t-RPS6 downregulation by the gefitinib+MK2206 treatment. Moreover, siRNA-based *RPTOR*-KD, but not *RICTOR* (rapamycin-insensitive companion of mTOR), downregulated the t-RPS6 protein and this reduction was potentiated by gefitinib addition, supporting the idea that mTORC1 controls the level of t-RPS6 in TNBC cells. RPTOR-dependent downregulation of t-RPS6 inhibited the proliferation of TNBC cells in the presence of gefitinib [451]. Although these results suggest RPS6 as an attractive therapeutic target to treat TNBC, the underlying mechanism maintaining the RPS6 level and its contribution to EGFRi resistance remain to be determined.

RPS6 has been reported to contribute to the regulation of intraductal colonization of basal-like breast cancer cells [457]. In an immortalized basal-like breast epithelial cell line, RPS6 dephosphorylation initiated the keratinization process. In addition, dephosphorylated RPS6 triggered detachment-induced keratin 5 (KRT5) upregulation post-transcriptionally. P-RPS6 was not detected in suspension cells in 3D cultures, whereas reconstitution of p-RPS6 by a constitutively active S6K mutant attenuated keratinization. Consistently, pharmacological inhibition of RPS6 phosphorylation caused sporadic keratinization in attached cells. Reduced RPS6 phosphorylation was also identified in a prolonged detached TNBC cell line, MDA-MB-468 cells, and pharmacological inhibition of p-RPS6 also caused keratinization in attached MDA-MB-468 cells [457].

#### 4.2.3. RPS6 in Gastric Cancer

AMPK negatively regulates mTORC1 activity through direct phosphorylation of TSC2 and RPTOR (Figure 2): (1) It phosphorylates and activates TSC2, which inhibits the mTORC1 kinase activity [96,458]; and (2) it phosphorylates RPTOR, leading to its binding to 14-3-3, which suppresses the mTORC1 activity [459]. High levels of p-RPS6 and low levels of p-AMPKα have been reported to be associated with gastric tumor progression and to be independent predictors of patient survival after resection of primary cancer [437]. The median OS values of 28 vs. 73 months and 28 vs. 78 months in patients with positive vs. negative expression of p-RPS6 and with negative vs. positive expression of p-AMPKα, respectively.

#### 4.2.4. RPS6 in Glioblastoma

High-level expression of RPS6 is strongly correlated with GBM stem cell (GSC) markers, Nestin and SRY-box transcription factor 2 (SOX2), and an oligodendrocyte progenitor cell marker, oligodendrocyte transcription factor 2 (OLIGO2), in GBM tissues [438]. High levels of RPS6 and SOX2 are detected in high-grade GMB samples compared with the levels in the low-grade samples. RPS6 may contribute to the stemness of GSC through activation of the JAK/STAT3 pathway to induce these stemness-related proteins (see Section 5.2.5. RPS6-KD in Glioblastoma Cells). *RPS6*-KD reduced p-JAK2 and p-STAT3 and suppressed the tumor sphere formation, a characteristic of GSCs, of GBM cells in vitro. Inversely, RPS6 overexpression facilitated the tumor sphere formation, and the STAT3 inhibitor, AG490, antagonized the RPS6-enhanced sphere formation.

#### 4.2.5. RPS6 in Head and Neck Cancer

In patients with HNSCC, double-positive p21 and p-RPS6 present better disease-specific survival [439]. The p21-p-RPS6 double-positiveness was determined to be mTORC1-dependent but not p53-dependent. The inhibitory phosphorylation of 4E-BP1 by mTORC1 stabilizes p21 by inhibiting its degradation that is induced by its interaction with 4E-BP1 [460]. Consistently, the PI3K/AKT/mTORC1 pathway is frequently activated in HNSCC [439].

An earlier study suggested that RPS6 phosphorylation is associated with early events of OSCC tumor progression [441]. Compared with healthy control samples (15/30; 50%), clinical samples of epithelial dysplasia (15/15; 100%) and OSCC (47/53; 88.68%) have displayed higher frequencies of p-RPS6 (S40/244). High-level expression of p-RPS6 has been also identified in OSCC clinical samples [442]. P-RPS6 is correlated with p21 expression and inversely correlated with the tumor size and local infiltration.

High levels of p-RPS6 (240/244) are associated with shorter disease-free survival (DFS) than low levels in patients with ESCC [436]. There was no difference in the OS rate. The association was more significant in the early-stage ESCC patients than in the late-stage patients. In addition, the high ratio of p-RPS6/t-RPS6 resulted in a more significant correlation with adverse prognosis than the p-RPS6 level alone [436]. Interestingly, *RPS6*-KD resulted in anticancer effects in ESCC cell lines (see Section 5.2.3. RPS6-KD in Head and Neck Cancer Cells).

#### 4.2.6. RPS6 in Lung Cancer

The positivity of RPS6 and p-RPS6 (S235/236) is significantly higher in NSCLC clinical samples than in normal tissues (82.4% vs. 55.8% and 62.6% vs. 53.3%, respectively) [37]. It has been correlated with shorter median OS and DFS in NSCLC patients with high p-RPS6 levels than in patients with low levels (10 months vs. 60 months and 21 months vs. 48 months, respectively). However, although the positiveness of t-RPS6 showed differences, they did not reach statistical significance (30 months vs. 43 months and 26 months vs. 40 months, respectively; *p* > 0.05) [37]. In a separate study, hyperphosphorylation of RPS6 (S235/236) or the ratio of p-RPS6 to t-RPS6 was reported to be a predictive marker for survival of patients with NSCLC [38]. From the analysis of patient samples, the 5-year survival rate and median OS of patients with high levels of p-RPS6 were found to be significantly lower than those with p-RPS6(-) (3.0% vs. 26.5%; 20 months vs. 42 months, respectively). The significance was greater in patients with a high p-RPS6/t-RPS6 vs. patients with a low p-RPS6/t-RPS6 (2.1% vs. 32.0%; 12 months vs. 48 months, respectively) [38]. The predictability of the p-RPS6/t-RPS6 ratio was more powerful in patients of stage I NSCLC (8 months vs. 61 months). In addition, hyperphosphorylation of RPS6 is correlated with unfavorable clinical survival outcomes in NSCLC, lung adenocarcinoma, and GC [38,437,461].

#### 4.2.7. RPS6 in Melanoma

The suppression of p-RPS6 has been identified as a predictive marker for improved progression-free survival (PFS) in *BRAF*-mutant melanoma treated with RAF or MEK inhibitors. A study conducted sensitivity assays of growth inhibition and apoptosis induction for the RAF inhibitor (RAFi), vemurafenib, and the MEK inhibitor (MEKi), selumetinib, against a panel of 16 *BRAF*-mutant melanoma cell lines. The results suggested that the inhibition of p-ERK is necessary but not sufficient to predict the sensitivity of melanoma cell lines to the RAFi or MEKi [440]. Further analysis revealed that the sensitivity of melanoma cell lines to these drugs correlates well with the decrease in the levels of p-RPS6 (S235/236). Resistant cell lines maintain the p-RPS6 levels after RAFi or MEKi treatment. The degree of suppression of RPS6 phosphorylation was a predictable indication of the sensitivity to these drugs not only in xenograft models but also in melanoma patients in a prospective evaluation. Although the number of patients was small (reduction vs. no reduction, *n* = 6 vs. *n* = 3, respectively), the reduction of p-RPS6 levels after treatment of the RAFi vemurafenib was correlated with an approximately 5-fold improvement in PFS [440].

#### 4.2.8. RPS6 in Ovarian Cancer

High levels of RPS6 are associated with poor OS and PFS in OEC [40]. The RPS6 protein level was evaluated in clinical tissue samples, and poor clinical outcomes were found with median OS values of 30 vs. 42 months and median PFS values of 15 vs. 21 months in patients with high vs. low RPS6 levels, respectively [40]. High levels of p-RPS6 and NOTCH3 are associated with poor prognosis and a shorter OS (12.3 months) than the OS (81.9 months) of patients with low p-RPS6 and NOTCH3 levels in OEC [443]. Although NOTCH is known to activate the PI3K/mTORC1 pathway, the details are still largely unknown [462]. In addition, the roles of RPS6 in conjunction with NOTCH-related cancers remains to be determined.

#### 4.2.9. RPS6 in Renal Cell Carcinoma (RCC)

In RCCs, RPS6 has been identified as the key mediator of the antitumor effects of everolimus (RAD001), a rapalog [445]. Everolimus binds to the 12 kDa FK506-binding protein (FKBP12) to form a complex, which binds to mTOR to destabilize and inactivate the mTORC1 complex [348,463]. Everolimus reduces the clonogenicity and proliferation of RCC cells by blocking protein biosynthesis but does not induce apoptosis. Knockdown of *RPS6*, but not *EIF4EBP1* (the gene encoding 4E-BP1) or *CDKN1B*, abolishes the everolimus-induced anti-proliferative effect and blocks translation. More importantly, high levels of RPS6 and p-RPS6 have been found inversely correlated with the survival of patients with RCC [445].

#### 4.2.10. RPS6 in Vulvar Squamous Cell Carcinoma (VSCC)

The non-viral-associated VSCC results from genetic and epigenetic changes in the absence of papillomavirus infection. Genetic alterations in the differentiated vulvar intraepithelial neoplasia (VIN) include allelic imbalance, microsatellite instability, and *TP53* (the gene encoding p53) mutations [464,465]. P-RPS6 (S235/236)-positiveness has been identified in VSCC and differentiated VINs but not in basal and parabasal cells of the healthy vulvar epithelium. Additionally, laminin γ^2^ expression has solely been identified within p-RPS6 positive regions [447]. The expression of laminin γ^2^ is not detectable via immunohistochemical analysis in the normal epithelial basal cells, whereas its expression is a well-established marker of carcinoma [466,467,468].

### 4.3. RPS6 in Anticancer Drug Resistance

As mentioned earlier, the overexpression of RPS6 has been implicated in intrinsic or acquired drug resistance of cancer cells (see Section 3.2.4. Regulation of Drug Resistance) [39,352,353,354,355,356,357,358,359,360]. More importantly, constitutive phosphorylation of RPS6 has been found to confer drug resistance in breast cancer cells, GC cells, and melanoma cells [39,359,360].

#### 4.3.1. RPS6 in Resistance to HER2 Inhibitors (HER2is)

The level of p-RPS6 in trastuzumab-resistant HER2-positive (+) breast cancer cells inversely correlated with their susceptibility to HER2-targeting drugs [360]. Trastuzumab (Herceptin^®^), a US FDA-approved drug, is a humanized monoclonal antibody against HER2 to treat HER2+ breast cancer [469]. However, the benefit of trastuzumab is limited by intrinsic or acquired resistance [470,471]. The phospho-proteins in the PI3K/mTORC1 pathway, including p-mTOR (S2448), p-AKT (S473), and p-RPS6 (S235/236), have been tested in response to trastuzumab in both sensitive and resistant HER2+ breast cancer cells [360]. Among these proteins, the p-RPS6 level and the degree of its response to the treated drugs could predict the susceptibility of breast cancer cells to not only trastuzumab but also other drugs, including rapamycin, AZD2014, BEZ235, erlotinib, lapatinib, MK226, and OSI-906. Drugs that could not downregulate p-RPS6, such as trastuzumab, erlotinib, MK2206, and OSI-906, failed to inhibit the growth of trastuzumab-resistant breast cancer cells [360]. Moreover, the combination of trastuzumab and lapatinib further reduced the p-RPS6 level, alongside concurrent synergistic inhibition of cell growth. These results suggested the p-RPS6 level as a predictive marker for the susceptibility of HER2+ breast cancers to anti-HER2 drugs and a feasibility marker of drugs for a combination strategy for the trastuzumab-resistant HER2+ breast cancers [360].

#### 4.3.2. RPS6 in Resistance to MEK Inhibitors (MEKis)

Selumetinib (Koselugo^TM^) is a very recently FDA-approved MEK1/2 inhibitor for patients with neurofibromatosis [472]. Anticancer activity of selumetinib has been tested in a panel of 12 CRC cell lines [353]. The study found that the levels of p-S6K1 (T3889) and p-RPS6 (S235/236) remained unaffected or increased by selumetinib treatment in cell lines with intrinsic resistance. Resistant primary tumors from patients also demonstrated a similar response. Pharmacological inhibition of the PI3K/AKT/mTORC1/S6K1 pathway or *S6K1*-KD overcame the selumetinib resistance in these CRC cells [353].

The phosphorylation level of mTORC1 downstream effectors, such as S6K1, 4E-BP1, and RPS6, has been found to be associated with resistance to the MEKi PD0325901 in a panel of 48 GC cell lines [359]. Interestingly, MEKi sensitivity was independent of the level of p-ERK suppression but dependent on the degree of suppression in the levels of p-S6K1 (T389), p-4E-BP1 (S65), and p-RPS6 (S235). On the contrary, no significant suppression of phosphorylation in these proteins was observed in resistant cells after MEKi treatment. Among them, the change in p-RPS6 level by MEKi showed the strongest correlation with MEKi sensitivity in vitro. This resistance of GC cells to MEKi was similarly recapitulated in xenograft models [359]. In addition, since the p-AKT level in sensitive GC cells was not affected by MEKi, the MEKi-dependent suppression of p-RPS6 was suggested to be mediated by mTORC1 in these cells. Interestingly, ERK regulates the level of p-RPS6 not only through RSK but also the TSC1/2 complex, the upstream negative regulator of mTORC1: (1) ERK1 induces inhibitory phosphorylation of TSC2 [74]; (2) ERK also phosphorylates and activates S6K [73]; and (3) ERK activates mTORC1 through RSK-mediated RPTOR phosphorylation (Figure 2) [75]. Previously, p-RPS6 has been demonstrated to be a potential predictive marker for the responsiveness of RCC cells to everolimus [445]. Taken together, further evaluation is warranted for RPS6 and/or p-RPS6 as a companion diagnostic for the therapeutic use of various inhibitors for mTOR and MEK.

#### 4.3.3. RPS6 in Resistance to RAF Inhibitors (RAFis)

Currently, BRAF^V600E^-selective inhibitors (BRAFis), such as dabrafenib, encorafenib, and vemurafenib, and MEK inhibitors (MEKis) including binimetinib, cobimetinib, and trametinib, have been approved by the US FDA for the treatment of melanoma [213]. However, similar to other clinical PKIs, acquired resistance to these inhibitors is rapidly developed after a transient clinical benefit [473]. The constitutive phosphorylation of RPS6 has been found in *BRAF*^V600E^-mutant melanoma cell clones with acquired resistance to PKIs [39]. In two human melanoma clones, A375-DR and A375-TR, with resistance to the BRAFi, dabrafenib, and the MEKi trametinib, respectively, p-RPS6 could not be downregulated by other MAPK pathway inhibitors. In contrast, an S6K inhibitor LY2584702 downregulated p-RPS6 in these cells. Additionally, the PI3K/mTOR inhibitor BEZ235 and the mTOR inhibitor AZD2014 could also inhibit the RPS6 phosphorylation in the resistant cells. Therefore, the switched regulation of p-RPS6, from the RAS/RAF/MEK/ERK pathway to the PI3K/AKT/mTORC1/S6K pathway, led to the resistance of these cells to BRAFis and MEKis by inducing the expression of G_0_/G_1_ cell cycle checkpoint proteins, including RB, cyclin D1, and CDK6 [39]. The mechanism underlying this mechanistic switched regulation of p-RPS6 remains to be evaluated.

#### 4.3.4. RPS6 in Resistance to PARP Inhibitors (PARPis)

The poly(ADP-ribose) polymerase (PARP) is a well-established target to treat cancers with HRDs, including mutations in *BRCA1/2* (breast cancer susceptibility gene 1/2) [474]. Currently, four small-molecule PARPis, olaparib (Lynparza^®^), talazoparib (Talzenna^®^), rucaparib (Rubraca^®^), and niraparib (Zejula^®^), have been approved by the US FDA to treat breast, ovarian, pancreatic, and prostate cancer [475]. P-RPS6 has been reported to confer the *BRCA1*-deficient breast cancer HCC1937 cells with resistance to the PARPi olaparib [376]. Long-term treatment of HCC1937 cells with olaparib upregulated p-RPS6, whereas restoration of BRCA1 abrogated the olaparib-induced RPS6 phosphorylation. In addition, RPS6^P−/−^ breast cancer cells with a *BRCA1*-defective background (HCC1937) demonstrated olaparib sensitivity. More interestingly, in olaparib-resistant HCC1937 cells, IR decreased and increased γ-H2AX foci and RAD51 foci, respectively, whereas IR elicited exactly the opposite effects in RPS6^P−/−^ HCC1937 cells. Since γ-H2AX is a surrogate marker of DNA DSBs, and RAD51 is a marker of HR, these results imply that p-RPS6 plays a critical role in inducing HRR to eliminate DSBs in *BRCA1-*deficient cells. More importantly, blocking phosphorylation of RPS6 by rapamycin or BEZ235 in combination with olaparib results in synergistic anticancer activity both in vitro and in vivo [374,376].

## 5. RPS6 as a Therapeutic Target in Cancer

Substantial evidence suggests that RPS6 is a potential therapeutic target against cancers. Knockdown experiments have demonstrated that RPS6 itself, not only p-RPS6, is indispensable for the proliferation or survival of various cancer cells (Table 9).

### 5.1. MicroRNAs Targeting RPS6

*MiR-129-5p* sensitized HER2(+) breast cancer to trastuzumab (Herceptin) by targeting *RPS6* [476]. The level of *miR-129-5p* is reduced in the trastuzumab-resistant human breast cancer cells, JIMT-1, and patient serum samples [476,481]. *MiR-129-5p* reduced RPS6 expression by targeting the 3′-untranslated region (3′-UTR) of *RPS6* mRNA, and transient transfection of the miR-129-5p mimic increased the trastuzumab sensitivity of JIMT-1 cells via downregulating RPS6 expression [476]. Consistent with this observation, the inhibition of *miR-129-5p* in the trastuzumab-susceptible breast cancer cell line, SK-BR-3, induced trastuzumab resistance, whereas concurrent treatment of *RPS6*-siRNA abolished this resistance. Similarly, the *miR-129-5p* overexpression-induced trastuzumab-sensitivity was revered by RPS6 overexpression in JIMT-1 cells [476].

### 5.2. RPS6-KD in Cancer Cells

**Figure 5:**
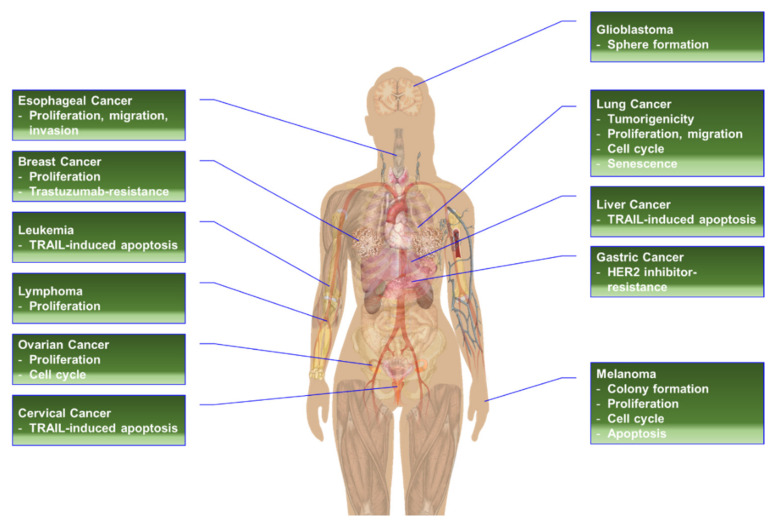
The effects of *RPS6*-KD in cancer cells.

RPS6 or its phosphorylation has been proposed as an alternative target to treat cancer types with high levels of RPS6 [21,482] (Figure 5). The siRNA- or shRNA-based *RPS6*-KD has demonstrated anticancer effects in various cancer cells (Table 9).

#### 5.2.1. RPS6-KD in Breast Cancer Cells

Studies on the TNBC cells HS578T and MDA-MB-231 have suggested RPS6 as a therapeutic target against TNBC [21]. Synergistic downregulation of both p-RPS6 (S235236) and t-RPS6 has been identified as a unique feature in TNBC cells co-treated with the EGFRi gefitinib and the METi SU11274, whereas no significant or consistent changes have been observed in the levels of p-EGFR (Y1068), t-EGFR, t-MET, p-AKT (S473), t-AKT, p-ERK1/2 (T202/Y204), and t-ERK1/2. Additionally, 4 h treatment with the proteasome inhibitor MG132 was not sufficient to inhibit the reduction of t-RPS6 in the TNBC cells in the presence of gefitinib and SU11274. The siRNA-based *RPS6*-KD reproduced the reduction of cell proliferation observed in TNBC cells co-treated with the gefitinib alongside SU11274. Unfortunately, the functional consequence of *RPS6*-KD in TNBC cells has not been further studied. However, it is noteworthy that the downregulation of t-RPS6 was augmented over time, whereas the initial reduction in the levels of p-EGFR and p-AKT was recovered with time in the TNBC cells co-treated with gefitinib and SU11274 [21]. Further research is needed to address the mechanism of regulating RPS6 stability in TNBC cells and its implication for TNBC treatment. *RPS6*-KD or rapamycin treatment has been reported to upregulate p-4E-BP1 (T37/46) and acetylated histone H3 (Ac-H3) in TNBC cells [477]. Co-treatment of the cells with valproic acid (VA) and tamoxifen suppresses *RPS6*-KD-mediated induction of p-4E-BP1 and Ac-H3 (K56) levels, and the triple combination of rapamycin, VA, and tamoxifen suppresses p-RPS6 (S235/236), p-4E-BP1 (T37/46), and Ac-H3 (K56) levels concurrently with a decrease in the viability of TNBC cells. Interestingly, this triple combination restored the expression of estrogen receptor alpha (ERα). In addition, both the triple combination and *RPS6*-KD with VA and tamoxifen reduced the cancer stem cell (CSC) population in TNBC cells. Furthermore, the triple combination reduced tumor growth, CSC subpopulation, and tumorigenesis in vivo [477].

#### 5.2.2. RPS6-KD in Cervical Carcinoma Cells

Downregulation of RPS6 in the cervical carcinoma cell line HeLa resulted in the inhibition of TRAIL-dependent apoptosis in a DR4-dependent manner [340]. *RPS6*-KD downregulated DR4 and desensitized these cancer cells to TRAIL-dependent apoptosis (see Section 5.2.7. RPS6-KD in Hematopoietic Cancer Cells). Conversely, the overexpression of DR4 sensitized TRAIL-induced apoptosis in *RPS6*-KD cells. The detailed mechanism underlying the regulation of DR4 expression by RPS6 remains to be described. Similar to Jurkat cells expressing antisense *RPS6*, HeLa cells stably expressing antisense *RPS6* or *RPS6*-shRNA were not resistant to apoptosis induced by doxorubicin or tunicamycin. In contrast, RPS6 overexpression in HeLa cells resulted in sensitization of the cells to TRAIL-dependent apoptosis, but not to apoptosis induced by doxorubicin, etoposide, staurosporine, or tunicamycin. Overexpression of other RPSs, such as RPS2, RPS3, and RPS20, did not affect TRAIL-induced apoptosis in HeLa cells. As mentioned earlier (see Section 3.2.3. Regulation of Apoptosis), the sensitization of TRAIL-induced apoptosis is mediated by unphosphorylated RPS6 not by p-RPS6 in HeLa cells.

#### 5.2.3. RPS6-KD in Head and Neck Cancer Cells

The level of p-RPS6 (S240/244) and the ratio of p-RPS6/t-RPS6 are correlated with unfavorable prognosis in ESCC patients [436]. The important role of RPS6 in ESCC cells was further demonstrated by a knockdown experiment. *RPS6*-KD in the ESCC cell lines TE8 and TE10 reduced the numbers of these cells over time compared with the numbers of the control KD cells. Western blot analyses showed downregulation of cyclin D and CDK2 proteins in the *RPS6*-KD ESCC cells. In contrast, the levels of p21 and p27 were induced by *RPS6*-KD. Furthermore, *RPS6*-KD reduced the migration, invasion, and focal adhesion formation of ESCC cells, concurrently with a reduction of p-FAK (Y397), p-paxillin (Y118), p-ERK, and p-JNK (T183/Y185). *S6K1*-KD, an upstream kinase of RPS6, also resulted in similar effects in ESCC cells [436].

#### 5.2.4. RPS6-KD in Gastric Cancer Cells

As mentioned earlier, *RPS6*-KD, in HER2i-resistant GC cell lines, resulted in a reduction in viability and induced sensitivity to HER2is through the downregulation of NRF2 and its target genes such as *AKR1C1*, *AKR1C2*, and *AKR1B10* [354] (see Section 3.2.4. Regulation of Drug Resistance). The mechanisms of NRF2 downregulation by *RPS6*-KD remain largely unknown. Notably, RPS6 has been reported to bind to the La autoantigen (also known as Sjögren syndrome type B; SSB) upon oxidative stress, leading to increased translation of NRF2 [370]. La/SSB binds to the 5′-UTR of *NRF2* mRNA in response to oxidative stress, and RPS6 consequently associates with the ribosomes.

#### 5.2.5. RPS6-KD in Glioblastoma Cells

*RPS6*-KD suppressed the tumor-sphere formation of GBM cells through the inhibition of p-JAK2 and p-STAT3 and concomitantly downregulated the stemness-related proteins Nestin and SOX2 [438]. The size of the tumor spheres was also reduced by *RPS6*-KD. Inversely, transient overexpression of RPS6 enhanced the tumor-sphere formation of these cells [438]. Furthermore, the interleukin 6 (IL6)-induced tumor sphere formation was reduced by *RPS6*-KD. Taken together, RPS6 may regulate the IL6/IL6 receptor (IL6R) pathway. Since the tumor sphere formation and the expression of Nestin and SOX2 are the characteristics of GSCs, these results suggest that RPS6 plays critical roles in the development and maintenance of GSCs [438]. Interestingly, the stemness-related proteins Nestin and SOX2 have been established as transcriptional targets of STAT3 [483,484].

#### 5.2.6. RPS6-KD in HCC Cells

Similar to HeLa cells (see Section 5.2.2. RPS6-KD in Cervical Carcinoma Cells), *RPS6*-KD in the human HCC cell line SK-HEP-1 suppressed TRAIL-induced apoptosis and downregulated DR4 [340]. The reconstitution of DR4 expression rescued the TRAIL-resistance in this cell line.

#### 5.2.7. RPS6-KD in Hematopoietic Cancer Cells

A functional genetic screening for the death-rescue modifier has identified TRAIL-resistant T cell leukemia Jurkat clones that contain antisense *RPS6* cDNA in their genome [340]. The reduced RPS6 expression in these cells resulted in the attenuation of TRAIL-induced apoptosis. This resistance was specific against TRAIL. No attenuation was observed in cell death caused by other signals, such as tumor necrosis factor-α (TNFα), FAS ligand (FASL), etoposide, and staurosporine.

As previously mentioned, *RPS6*-KD reduces the proliferation of the PEL cell line BC-3 (see Section 3.2.10. Roles of RPS6 in Response to Infection) [399]. Since BC-3 cells were infected by KSHV, the cells contain the viral genome and express the viral proteins as well [485]. RPS6 may contribute to the extraordinary stability (several days) of LANA. *RPS6*-KD remarkably reduced the LANA stability to 0.6 hr, leading to p53 stabilization and a p53-target gene, *CDKN1A* (the gene encoding p21) expression [399]. However, the molecular mechanism of RPS6 in the LANA stabilization needs further study.

*RPS6*-KD has decreased the levels of the proliferating cell nuclear antigen (PCNA) in p53 wild-type DLBCL cell lines [338]. However, *RPS6*-KD did not induce apoptosis in these cells.

#### 5.2.8. RPS6-KD in Lung Cancer Cells

The suppression of proliferation by shRNA-based *RPS6*-KD was exaggerated by time in two lung cancer cell lines, A549 and H520 [37]. Inversely, the expression of senescence-associated β-galactosidase (SA-β-gal) was increased by *RPS6*-KD in both cell lines. Additionally, *RPS6*-KD increased the G_0_/G_1_ phase and decreased the G_2_/M phase with reduced protein levels of p-RB and cyclin D1, whereas no significant changes were observed in the levels of cyclin A, cyclin E, and total RB. More interestingly, the levels of CKIs, including p16, p21, p27, and p57, were increased. However, *RPS6*-KD did not induce apoptosis or altered the expression of BCL-xL, BAX, or caspase-3. The anticancer effect of *RPS6*-KD was reproduced in a xenograft model in which A549 lung cancer cells with *RPS6*-KD resulted in reduced tumorigenicity with an increase in the number of SA-β-gal(+) cells in the xenograft tissues. Similar changes were observed in the expression of cell cycle regulators and CKIs [37].

*RPS6*-KD induced the G_0_/G_1_ cell-cycle arrest, decreasing cell viability in the NSCLC cell lines SK-MEK-1 and H1650 [38]. The levels of p-RB, cyclin D1, cyclin E, CDK2, and CDK4 were also reduced, whereas the levels of CKIs, p21, and p27 were increased by *RPS6*-KD in these cells. The migration of NSCLC cells was also reduced by *RPS6*-KD, which also downregulated the proteins involved in cell migration, including N-cadherin, vimentin, MMP-9, MMP-2, and p-paxillin. In contrast, E-cadherin was upregulated by *RPS6*-KD. Inversely, the overexpression of RPS6 in a normal HBE cell line promoted cell proliferation concurrently with the upregulation of p-RPS6, p-RB, cyclin D1, cyclin E, CDK2, and CDK4, and decreases in CKIs and the number of cells in the G_0_/G_1_ phase. The migration of HBE cells was also enhanced by RPS6 overexpression. Additionally, RPS6 overexpression upregulated N-cadherin, vimentin, MMP-2, and p-paxillin in these cells [38].

As mentioned earlier (see Section 3.2.1. Regulation of Cell Cycle, Proliferation, and Growth), *RPS6*-KD by siRNA in A549 cells induced the p53-mediated checkpoint in an RPL11-depenent manner [339]. Although *RPS6*-siRNA transfection was not enough to completely deplete the RPS6 protein due to the long half-life of ribosomes, it induced the expression of p53 and p21, leading to a partial decrease in DNA synthesis and an increase in the number of cells arrested at the G_1_ phase. The concurrent depletion of *TP53* by siRNA abrogated the effects of *RPS6*-siRNA transfection. *RPS6* depletion increased the translation of *RPL11* mRNA and the amount of ribosome-free RPL11, which can bind to MDM2, leading to the inhibition of MDM2 function to degrade p53. Consequently, the mRNAs and protein levels of p53 targets, such as *CDKN1A* (the gene encoding p21) and *MDM2,* were increased.

#### 5.2.9. RPS6-KD in Melanoma Cells

*RPS6*-KD alone reduced the proliferation of two human melanoma cells A375-DR and A375-TR, which are resistant to the BRAF^V600E^-selective inhibitor dabrafenib and the MEK1/2 inhibitor trametinib [39]. In addition, *RPS6*-KD induced the G_0_/G_1_ cell-cycle arrest in these cells. More interestingly, *RPS6*-KD was sufficient to reduce the levels of G_0_/G_1_ checkpoint regulators such as RB, cyclin D1, and CDK6. Additionally, the enhanced downregulation of p-RPS6 by drug combinations exerted a synergistic antitumor activity [39]. 

In a separate study, *RPS6*-KD significantly reduced the colony formation and induced the apoptosis of cutaneous malignant melanoma (CMM) cells in vitro [478]. Additionally, the same authors found that S6K1 was upregulated in clinical samples with advanced stages of CMM. Furthermore, the mTORC1 signaling pathway was identified to be downregulated by the combination of the EGFRi afatinib and the ALK/ROS1 dual inhibitor crizotinib in malignant melanoma. More interestingly, the level of t-RPS6 itself, in addition to p-RPS6 level, was also downregulated by the afatinib+crizotinib treatment in melanoma cell lines in vitro [478].

#### 5.2.10. RPS6-KD in Ovarian Cancer Cells

It has been reported that the transfection of ovarian cancer cells with *RPS6*-siRNA reduces the activities of glutaminase (GLS) and glutamate dehydrogenase (GDH), leading to a reduction in glutamine-induced proliferation of the cells [479]. Since glutamine is the main nutrient used by cancer cells, targeting the glutamine metabolism has been recognized as a promising therapeutic alternative [486]. Glutamine depletion inhibits cell proliferation concurrently with the induction of G_1_ cell-cycle arrest and apoptosis in ovarian cancer cells [479]. Glutamate increases cell proliferation in the presence of glucose. Interestingly, rapamycin reduced the glutamine-induced p-RPS6 (S235/236) and GLS expression in a dose-dependent manner in ovarian cancer cells. In the ovarian cancer cell line HEY, transfection of *RPS6*-siRNA was sufficient to deplete p-RSP6 (S235/236) although not t-RPS6 [479]. Under these conditions, *RPS6*-siRNA transfection reduced the glutamine-induced GDH activity and cell proliferation in HEY cells, whereas no significant effect of *RPS6*-siRNA transfection was observed in the absence of glutamine. Notably, rapamycin treatment only partially mimicked the effects of *RPS6*-siRNA transfection. The significance of this discrepancy remains to be elucidated.

*RPS6*-KD also inhibits the proliferation and long-term colony formation of the ovarian cancer cell lines SKOV-3 and HO-8910 [40]. The migration and invasion of these ovarian cancer cells are also reduced by *RPS6*-KD. Additionally, *RPS6*-KD induces the G_0_/G_1_ cell-cycle arrest. Consistently, the G_0_/G_1_ checkpoint regulators, such as cyclin D1, cyclin E, CDK2, CDK4, CDK6, and p-RB, are diminished by *RPS6*-KD in ovarian cancer cells [40].

#### 5.2.11. RPS6-KD in Sarcoma Cells

An siRNA-based screening identified RPS6 as an effector of IGF1R inhibition in sarcoma cells [480]. *RPS6*-KD by siRNA reduced the survival of Ewing sarcoma cell lines over time. Interestingly, *MTOR*-KD recapitulated *RPS6*-KD, whereas *TSC1*-KD had little or no effect. In addition, *RPS6*-KD also induced cell death in rhabdomyosarcoma (RMS) cell lines [480]. The significance of p-RPS6 in these sarcoma cell lines is underlined by their sensitivity to the IGF1R inhibitor BMS-536924. RMS cell lines were resistant to BMS-536924 compared to Ewing sarcoma cell lines. BMS-536924 failed to downregulate p-RPS6 (S235/236) and p-4EBP1 in RMS cell line, but dose-dependently downregulated p-RPS6 (S235/236) and p-4EBP1 in sensitive Ewing sarcoma cell lines. Additional siRNA screening has found that the knockdown of macrophage stimulating 1 receptor (MST1R) sensitizes RMS cell lines to BMS-536924 with concordant reduction of p-RPS6 (S235/236). These data suggest that MST1R may be an alternative activator of RPS6 in IGF1R inhibitor-resistance sarcoma cells.

## 6. Conclusions

**Figure 6:**
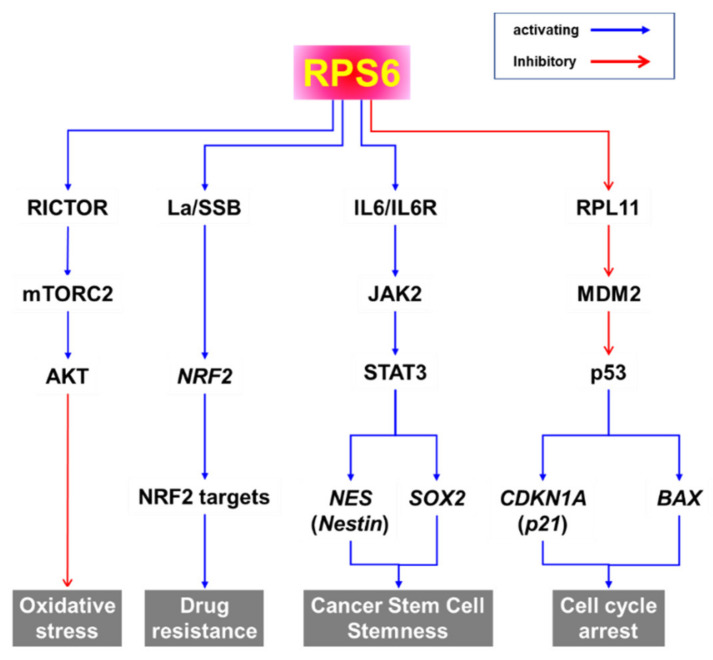
Putative signaling pathways downstream of RPS6.

In addition to its role in protein synthesis, RPS6 may activate various pathways to induce the oxidative stress response, drug resistance, cancer stem cell stemness, and cell-cycle arrest (Figure 6). Evidence from knockdown experiments supports the therapeutic potential of RPS6 targeting in various cancer cells including breast cancer, cervical carcinoma, head and neck cancer, gastric cancer, glioblastoma, HCC, hematopoietic cancer, lung cancer, melanoma, ovarian cancer, and sarcoma (Figure 5). However, the signaling pathways downstream of RPS6 are currently largely unknown. In addition, the roles of activated RPS6 in tumorigenesis still remain elusive. The positive and negative feedback and/or feedforward mechanisms and novel regulatory pathways controlled by RPS6 may provide alternative therapeutic interventions to overcome the current limitations in cancer treatment.

## Figures and Tables

**Table 1:** Selected examples of the extracellular proteins regulating RPS6 phosphorylation.

Protein	Effects
BDNF (brain-derived neurotrophic factor)	✓ induces p-RPS6 (S240/244) through the PI3K/AKT/mTORC1/S6K pathway in rat cortical neurons [80]
C3	✓ induces p-RPS6 and p-S6K1 (S371) in Paneth cells from mice with acute gastrointestinal injury [81]
CG (chorionic gonadotropin)	✓ induces p-RPS6 (S235/236) through the PI3K/AKT/mTORC1/S6K pathway [82]
EGF (epidermal growth factor)	✓ induces p-RPS6 in serum-starved HEK293 cells [83]
FGF8 (fibroblast growth factor 8)	✓ induces p-RPS6 in serum-deprived condition in hepatocellular carcinoma (HCC) cells [84]
FGF18 (fibroblast growth factor 18)	✓ induces p-RPS6 in serum-deprived condition in HCC cells [84]
Insulin	✓ induces p-RPS6 (S235/236 and S240/244) in HEK293 cells [85]
IFNα (interferon α)	✓ induces p-RPS6 (S235/236) in a PI3K and mTOR-dependent manner in BCR-ABL expressing cells [86]
IFNα2 (interferon α2)	✓ decreases p-RPS6 (S240/242) at 24 and 48 h post treatment by activating RPS6KB in a STAT2-and dose-dependent manners in Daudi cells, but not in other examined cell lines including HeLa S3, MDA-MB-231, T98G, A549, U937, and BJAB [87]
IFNβ (interferon β)	✓ induces p-RPS6 (S235/236) in a PI3K- and mTOR-dependent manner in BCR-ABL expressing cells [86] ✓ decreases p-RPS6 (S240/242) at 24 and 48 h post treatment through activating RPS6KB in Daudi cells [87]
IFNν (interferon ν)	✓ induces p-RPS6 (S240/244) in either MEK/ERK- or S6K1/2-dependent manner [88]
MK (midkine)	✓ induces p-RPS6 (S235/236) in cell lines with low MK expression, including SH-SY5Y MK-KD, SH-EP, and L28A-N-5, but not in the MK high cell lines SH-SY5Y WT and SK-N-DZ [89]
NGF (nerve growth factor)	✓ induces p-RPS6 (S235/236) in rat pheochromocytoma PC12 cells [90]
OSM (oncostatin M)	✓ induces p-RPS6 (S235/236) in cervical cancer cells [91]
PDGF-BB (platelet-derived growth factor-BB)	✓ induces p-RPS6 (S235/236) through the AMPK/mTORC1 pathway in vascular smooth muscle cells [92]
Serum	✓ induces p-RPS6 (S235/236 and S240/244) through mTOR- and MEK-dependent mechanisms [48] ✓ increases p-RPS6 via S6Ks and translation of 5′-terminal oligopyrimidine track (5′-TOP) mRNAs in Swiss mouse 3T3 cells [93]
TNFα (tumor necrosis factor α)	✓ induces p-RPS6 (S235/236) by activating S6K [94,95]
WNT3A (WNT family member 3A)	✓ induces p-RPS6 (S240/244) and p-S6K (T389) in a manner independent on the β-catenin-dependent transcription [96]

**Table 3:** Selected examples of the membrane proteins regulating RPS6 phosphorylation.

Protein	Effects
GRPR (gastrin-releasing peptide receptor)	✓ contributes to p-RPS6 (S235/236 and S240/244) through the PI3K/AKT/mTORC1 pathway by suppressing PTEN expression in neuroblastoma [152]
PD-1 (programmed cell death protein 1)	✓ frequently overexpressed in MART-1^+^/CD45^−^ melanoma samples [149] ✓ Knockdown of *Pdcd1* (the gene encoding PD-1) reduces the growth of B16-F10 murine melanoma cells tumor spheres concurrently with downregulation of p-RPS6, whereas overexpression of *Pdcd1* upregulates p-RPS6 in vitro [149] ✓ binds to RPS6 and eIF-4E to promote their phosphorylation in the hepatocellular carcinoma cell line SMMC7721 [148]
PD-L1 (programmed death ligand 1; B7-H1, CD274)	✓ PD-L1 Ig (a recombinant PD-L1 Fc fusion protein) induces p-RPS6 and increases the 3D growth of B16-F10 murine melanoma cells in vitro, which is blocked by mTOR inhibitors (rapamycin or PP242) but not by PI3K inhibitors (wortmannin or LY294002) [149] ✓ PD-L1 expression is negatively correlated with p-RPS6 (S235/236) level in non-small cell lung cancer (NSCLC) clinical samples [150] ✓ Its stability is regulated through suppression of β-TrCP in an mTORC1/p70S6K-dependent manner in NSCLC cells [150] ✓ induced by *RPS6*-KD, *PTEN* mutations, and wortmannin, LY294002, AKT inhibitor III, or rapamycin in breast and prostate cancer cells, leading to immune-resistance [151]

**Table 4:** Selected examples of the transcription factors regulating RPS6 phosphorylation.

Protein	Effects
AR (androgen receptor)	✓ SiRNA-based *AR*-KD downregulates p-RPS6 (S235/236) in LNCaP prostate cancer cells [155]
BTF3 (basic transcription factor 3)	✓ *BTF3*-KD reduces total RPS6 in osteosarcoma SAOS-2 cells [156]
FOXO3 (Forkhead box protein O3)	✓ downregulates mTORC1/S6K/RPS6 pathway through transcriptional activation of *TSC1* [157]
FOXO17 (Forkhead box protein O17)	✓ induces p-RPS6, p-AKT (T308 and S473), and p-PDK1 in the human lung cancer cell line A549 [153]
MYC	✓ activates S6K activity to induce p-RPS6 via transcriptional suppression of *TSC2* gene [30]
mtp53^P151S^	✓ upregulates p-RPS6 (S240/244) by inhibiting AMPK signaling through direct binding to AMPKα subunit in head and neck squamous cell carcinoma (HNSCC) Tu138 cells [154]

**Table 6:** miRNAs regulating RPS6 level.

miRNA	Effects
miR-92b-3p	✓ miR-92b-3p inhibitor reduces both RPS6 mRNA and protein by direct targeting *Tsc1* in neonatal mouse ovaries [203]
miR-451	✓ promotes glioma cell proliferation and upregulates p-RPS6 (S235/236) through activating mTORC1 by targeting calcium-binding protein 39 (*CAB39*) that activates liver kinase B1 (LKB1) in normal glucose status in glioma cells [204] ✓ increased in colorectal tumor and its overexpression upregulates p-RPS6 (S235/235) through AMPK/mTOR signaling in a CRC cell line HT-29 [105]

**Table 7:** Selected examples of the natural ligands and stimuli regulating RPS6 phosphorylation.

Natural Ligand or Stimulus	Effects
Alpha-linoleic acid (ALA)	✓ downregulates p-RPS6 and upregulates p-AMPK, and reduces cell proliferation in esophageal cell carcinoma (ECC) cells [206]
Amino acids	✓ Leucine induces p-RPS6 (S235/236) in HEK293 cells [85] ✓ induces p-RPS6 (S240/244) through the mTORC1-S6K axis by activating MAP4K3 [116]
ATRA (all-*trans*-retinoic acid)	✓ stimulates an increase in ceramide, G_0_/G_1_ cell-cycle arrest, and suppresses p-RPS6 via inhibiting p-S6K in an nSMase2-dependent manner, but mTOR-, AMPK-, or GSK3β-independent manner in a breast cancer cell line MCF7 [184]
DHT (5α-dihydrotestosterone; androgen)	✓ induces p-RPS6 (S235/236) via the mTORC1/S6K pathway but not the PI3K/AKT, RSK, or AMPK pathway in LNCaP prostate cancer cells [207]
E_2_ (17β estradiol)	✓ induces p-RPS6 (S235/236) by decreasing the amount of inactive, GDP-bound RHEB [208]
Hydrogen peroxide (H_2_O_2_)	✓ induces p-RPS6 (S235/236) through stimulation of p85S6K1, but not p70S6K1, in an mTOR-independent manner in MCF7 and HCT116 cells [115] ✓ reduces p-RPS6 (S235/236) in the cytoplasm of MCF7 in a dose-dependent manner, potentially through the ATM/LKB1/AMPK/TSC2 pathway [205]
Hypoxia	✓ inhibits insulin-, leucine-, PMA-, and serum-induced p-RPS6 in HEK293 cells [85]
Mannitol	✓ downregulates p-RPS6 (S240/244) by inhibiting S6K [96]
Oleic acid (OA)	✓ downregulates p-RPS6 and upregulates p-AMPK, and reduces cell proliferation in esophageal cell carcinoma (ECC) cells [206]
Phosphatidic acid (PA)	✓ generated from phosphatidylcholine by phospholipase D 1/2 (PLD1/2) [209] ✓ directly and competitively binds to the rapamycin-binding domain of mTOR to stabilize mTORC1 and mTORC2 complex assembly [210,211] ✓ induces p-RPS6 via the mTORC1/S6K axis, and can rescue the mesalamine-induced reduction of p-RPS6 in CRC cells [212]

**Table 9:** Anticancer effects of *RPS6* depletion in human cancer cells.

Cancer Type	Cell Lines	Knockdown by	Effects of *RPS6*-KD	Ref
Breast cancer, trastuzumab-resistant	JIMT-1 SK-BR-3	miR-129-5p mimic/siRNA	✓ reduces the trastuzumab resistance in MTT cell viability assay	[476]
Breast cancer, triple-negative (TNBC)	HS578T MDA-MB-231	siRNA	✓ reduces cell proliferation over time	[21]
	MDA-MB-231 SUMM149PT	siRNA	✓ induces p-4E-BP1 and acetylated histone H3, whereas VA and tamoxifen treatment antagonize these effects	[477]
Cervical carcinoma	HeLa	Antisense & shRNA	✓ attenuates TRAIL-induced apoptosis ✓ downregulates DR4 ✓ no effect on the apoptosis induced by doxorubicin or tunicamycin	[340]
Esophageal squamous cell carcinoma (ESCC)	TE8 TE10	siRNA	✓ reduces cell proliferation, migration, and invasion ✓ downregulates cyclin D and CDK2 proteins ✓ upregulates p21 and p27 ✓ downregulates p-FAK (Y397), p-paxillin (Y118), p-ERK, and p-JNK	[436]
Gastric cancer, *HER2*-amplified	OE19 NCI-N87	siRNA	✓ reduces the expression of NRF2 and its target genes including *AKR1C1*, *AKR1C2*, and *AKR1B10* ✓ reduces the viability and resistance to HER2 inhibitors in vitro	[354]
Glioblastoma multiforme (GBM)	U251MG	siRNA	✓ decreases the sphere formation; transient overexpression of RPS6 increases the sphere-forming potential ✓ downregulates p-JAK2 and p-STAT3 ✓ abrogates the IL6-induced sphere formation ✓ downregulates stemness-related proteins Nestin and SOX2	[438]
Hepatocellular carcinoma (HCC)	SK-HEP-1	shRNA	✓ attenuates TRAIL-induced apoptosis ✓ downregulates DR4	[340]
Leukemia, T cell	Jurkat	antisense	✓ attenuates TRAIL-induced apoptosis ✓ No effect on the apoptosis induced by etoposide, FASL, staurosporine, or TNFα	[340]
Lung cancer (adenocarcinoma)	A549	siRNA	✓ increases RPL11 translation ✓ induces p53 protein and its transcriptional targets, including *CDKN1A (p21)*, *BAX*, and *MDM2* ✓ induces the p21 and MDM2 proteins	[339]
Lung cancer (adenocarcinoma)	A549	shRNA	✓ reduces cell proliferation ✓ induces the G_0_/G_1_ cell-cycle arrest, concurrently with reduced p-RB and cyclin D1 protein levels ✓ upregulates p16, p21, p27, and p57 ✓ upregulates SA-β-gal ✓ No detectable change in apoptosis ✓ reduces A549 tumorigenicity with increased SA-β-gal(+) cells in vivo	[37]
Lung cancer, non-small cell (NSCLC)	SK-MES-1 H1650	shRNA	✓ reduces the viability and migration ✓ induces the G_0_/G_1_ cell-cycle arrest concurrently with reduced levels of p-RB, cyclin D1, cyclin E, CDK2, and CDK4 proteins ✓ downregulates N-cadherin, vimentin, MMP-9, MMP-2, p-paxillin, but upregulates E-cadherin ✓ upregulates p21 and p27	[38]
Lung cancer (squamous cell carcinoma)	H520	shRNA	✓ reduces cell proliferation ✓ induces the G_0_/G_1_ cell-cycle arrest concurrently with reduced p-RB and cyclin D1 protein levels ✓ upregulates p16, p21, p27, and p57 ✓ upregulates SA-β-gal ✓ No detectable change in apoptosis	[37]
Lymphoma, primary effusion lymphoma (PEL)	BC-3	shRNA	✓ reduces cell proliferation ✓ induces p53 and p21	[399]
Lymphoma, non-Hodgkin	OCI-LY3 SUDHL-6	shRNA	✓ reduces proliferating cell nuclear antigen (PCNA) protein levels	[338]
Melanoma, cutaneous malignant (CMM)	A375 A375VR4 SK-MEL-2 3918	siRNA	✓ reduces colony formation in vitro ✓ induces apoptosis	[478]
Melanoma, cutaneous malignant (CMM), kinase inhibitor-resistant	A375-DR A375-TR	siRNA	✓ downregulates cyclin D1, CDK6, p-RB, and RB ✓ induces the G_0_/G_1_ cell-cycle arrest and inhibits cell proliferation	[39]
Ovarian cancer	HEY	siRNA	✓ reduces the glutamine-induced p-RPS6 (S235/235) but not total RPS6 ✓ reduces the glutamine-induced cell proliferation and glutamate dehydrogenase (GDH) activity	[479]
Ovarian cancer	SKOV-3 HO8910	siRNA	✓ reduces the proliferation, colony formation, migration, and invasion in vitro ✓ induces cell-cycle arrest at the G_0_/G_1_ phase ✓ reduces the expression of cyclin D1, cyclin E, CDK2/4/6, and p-RB	[40]
Sarcoma, Ewing	TC32 TC71	siRNA	✓ reduces cell survival	[480]
Sarcoma, alveolar Rhabdomyosarcoma	Rh18 Rh30	siRNA	✓ reduces cell survival	[480]

## Data Availability

Not applicable.

## Abbreviations

2-DG, 2-deoxyglucose; 4E-BP1, eukaryotic translation initiation factor 4E-binding protein 1; 5-ASA, 5-aminosalicylic acid; 5′-TOP, 5′-terminal oligopyrimidine track; 53BP1, p53-binding protein 1; ABL1, Abelson murine leukemia viral oncogene homolog 1; ADAR1, adenosine deaminase acting on RNA 1; AICAR, 5-aminoimidazole-4-carboxamide ribonucleotide; AIPS, autophagy impairment-induced premature senescence; AKR, aldo-keto reductase; AKR1B10, AKR family 1 member B10; AKR1C, AKR family 1 member C; ALA, alpha-linoleic acid; AML, acute myeloid leukemia; AMOG, adhesion molecule in glia; AMPK, 5′-AMP-activated protein kinase; AMPKK, AMPK kinase; AR, androgen receptor; ATG7, autophagy related 7; ATIC, AICAR transformylase/inosine monophosphate cyclohydrolase; ATM, ataxia telangiectasia mutated; ATRA, all-*trans*-retinoic acid; BAX, BCL2 associated X; BCL-xL, B-cell lymphoma-extra large; BDNF, brain-derived neurotrophic factor; β-TrCP, beta-transducin repeat containing protein; BRCA1, breast cancer type 1 susceptibility protein; BRD4, bromodomain 4; BTF3, basic transcription factor 3; CAB39, calcium-binding protein 39; CDK, cyclin-dependent kinase; CDKN1A, CDK inhibitor 1A; CDKN1B, CDK inhibitor 1B; C/EBP, CCAAT/enhancer binding protein; CG, chorionic gonadotropin; CHD3, chromodomain-helicase-DNA-binding protein 3; CK1, casein kinase 1; CKI, CDK inhibitor; CMM, cutaneous malignant melanoma; CRC, colorectal cancer; CREB, cAMP-responsive element-binding protein; CSC, cancer stem cell; DAPK1, death-associated protein kinase 1; DBA, Diamond-Blackfan anemia; DDB1, DNA damage-binding protein 1; DDIT4, DNA damage-inducible transcript 4 protein; DFS, disease-free survival; DHT, 5α-dihydrotestosterone; DLBCL, diffuse large B-cell lymphoma; DMBA, 7,12-dimethylbenz[a]anthracene; DNA-PK, DNA-dependent protein kinase; DR4, death receptor 4; DRAM1, DNA damage-regulated autophagy modulator protein 1; DRD1, dopamine D1 receptor; DSB, double-strand break; DYRK1A, dual-specific tyrosine-phosphorylation-regulated kinase 1A; E_2_, 17β estradiol; E2F1, E2F transcription factor 1; EGF, epidermal growth factor; EGFRi, EGFR inhibitor; eIF (3 and 4B), eukaryotic translation initiation factor (3 and 4B); ERα, estrogen receptor alpha; ERK, extracellular signal-regulated kinase; ESCC, esophageal squamous cell carcinoma; FASL, FAS ligand; FASN, fatty acid synthase; FBXO28, F-box-only protein 28; Fc, fragment crystallizable; FDA, Food and Drug Administration; FGF, fibroblast growth factor; FGFR, FGF receptor; FIP200, FAK family kinase-interacting protein of 200 kDa; FKBP12, 12 kDa FK506-binding protein; FOXO, Forkhead box protein O; FPT, farnesyl protein transferase; Gal-13, galetin-13; γ-H2AX, phosphorylated H2A histone family member X; GABP, GA-binding protein; GBM, glioblastoma multiforme; GDH, glutamate dehydrogenase; GlnRs, glutaminyl-tRNA synthase; GLS, glutaminase; GRPR, gastrin-releasing peptide receptor; GSK3β, glycogen synthase kinase-3β; HAP1, huntingtin-associated protein 1; HBE, human bronchial epithelial; HCC, hepatocellular carcinoma; HCV, hepatitis C virus; HDF, human dermal fibroblast; HER2, human EGFR 2; HER2i, HER2 inhibitor; HIF1α, hypoxia-inducible factor 1-alpha; HNSCC, head and neck squamous cell carcinoma; HRAS, Harvey rat sarcoma; HRR, homologous recombination repair; HRD, HRR deficiency; HSP90, heat shock protein 90; IGF1R, insulin-like growth factor 1 receptor; IFN, interferon; IFNAR2, interferon α/β receptor 2; IKAP, IκB kinase complex-associated protein; IKK, inhibitor of NF-κB kinase; IL (1 and 6), interleukin (1 and 6); IL6R, IL6 receptor; IPC, ischemic preconditioning; IR, ionizing radiation; IRES, internal ribosome entry site; KD, knockdown; KRAS, Kristen rat sarcoma; KRT5, keratin 5; KSHV, Kaposi’s sarcoma-associated herpesvirus; LAMP2, lysosome-associated membrane glycoprotein 2; LANA, latency-associated nuclear antigen; LCMT1, leucine carboxyl methyltransferase 1; LDHA, L-lactate dehydrogenase A chain; LKB1, liver kinase B1; LPIN1, lipin 1; mAb, monoclonal antibody; MAPK, mitogen-activated protein kinase; MAP4K3, mitogen-activated protein kinase kinase kinase kinase 3; MCD, multicentric Castleman’s disease; MDM2, mouse double minute 2; MEF, mouse embryonic fibroblast; MEK, MAPK-ERK kinase; MEKi, MEK inhibitor; METi, mesenchymal epithelial transition inhibitor; miRNA, microRNA; MK, midkine; MMP, matrix metallopeptidase; MST1R, macrophage-stimulating 1 receptor; mt, mutant; mTORC1, mechanistic/mammalian target of rapamycin complex 1; NAC, *N*-acetyl cysteine; NGF, nerve growth factor; NHDF, normal human dermal fibroblast; NLS, nuclear localization signal; NRF2, nuclear factor erythroid 2-related factor 2; NSCLC, non-small cell lung cancer; nSMase 2, neutral sphingomyelinase-2; OA, oleic acid; ORF45, open reading frame 45; OS, overall survival; OSCC, oral squamous cell carcinoma; OSM, oncostatin M; OEC, ovarian epithelial cancer; PA, phosphatidic acid; PALL, Pallbearer; PanIN, pancreatic intraepithelial neoplasia; PARP, poly (ADP-ribose) polymerase; PARPi, PARP inhibitor; PASK, PAS domain-containing serine/threonine-protein kinase; PCNA, proliferating cell nuclear antigen; PCNSL, primary central nervous system lymphoma; PD-1, programmed cell death protein 1; PDAC, pancreatic ductal adenocarcinoma; PDCD4, programmed cell death 4; PDGF, platelet-derived growth factor; PDK1, 3-phosphoinositide-dependent protein kinase 1; PD-L1, programmed death ligand 1; PEL, primary effusion lymphoma; PERK, protein kinase RNA-activated (PRKR)-like endoplasmic reticulum kinase; PFS, progression-free survival; PGC-1β, peroxisome proliferator-activated receptor-γ (PPAR-γ) coactivator-1β; PHLPP, pleckstrin homology domain leucine-rich repeat protein phosphatases; PI3K, phosphoinositide 3-kinase; PIM2, serine/threonine-protein kinase PIM2; PK (A, C, and G), protein kinase (A, C, and G); PKI, protein kinase inhibitor; PRKR, protein kinase RNA-activated; PLAC, placenta-specific 8; PLCG1, phospholipase C-γ1; PLD, phospholipase D; PLK, polo-like kinase; PMA, phorbol 12-myristate 13-acetate; PME1, protein phosphatase methylesterase 1; PP1, protein phosphatase 1; PP2A, serine/threonine-protein phosphatase-2A; PPAR-γ, peroxisome proliferator-activated receptor-γ; PPM1D, protein phosphatase magnesium-dependent 1 delta; PSIP1, PC4 and SFRS1-interacting protein 1; PTEN, phosphatase and tensin homolog; PyMT, polyomavirus middle tumor antigen; PyST, polyomavirus small tumor antigen; QARS, glutaminyl-tRNA synthase; RAC2, Rac family small GTPase 2; RACK1, receptor of activated PKC kinase 1; RAD51, DNA repair protein RAD51 homolog 1; RAF, v-raf-1 murine leukemia viral oncogene homology; RAFi, RAF inhibitor; RAME, rosmarinic acid methyl ester; RAS, rat sarcoma; RB, retinoblastoma-associated protein; RB1CC1, RB1-inducible coiled-coil protein 1; RCC, renal cell carcinoma; REDD1, regulated in development and DNA damage responses 1; RHEB, RAS homolog enriched in brain; RICTOR, rapamycin-insensitive companion of mTOR; RMS, rhabdomyosarcoma; ROS, reactive oxygen species; RP, ribosomal protein; RPL (5, 11, and 16), ribosomal protein L (5, 11, and 16); RPS (6, 8, and 24), ribosomal protein S (6, 8, and 24); rRNA, ribosomal RNA; RPTOR, regulatory-associated protein of mTOR; RSK, ribosomal protein S6 kinase; RVF, Rift Valley Fever; S6K, S6 kinase; SA-β-gal, senescence associated β-galactosidase; SASP, senescence-associated secretory phenotype; SCF, Skp-Cullin-F-box; SGK, serum and glucocorticoid-inducible kinases; SHE1, nucleoprotein sensitive to high expression protein 1; shRNA, short hairpin RNA; SIRT1, NDA-dependent protein deacetylase sirtuin-1; SOX2, SRY-box transcription factor 2; SSB, Sjögren syndrome antigen B; STK33, serine/threonine kinase 33; SP1, specificity protein 1; SQSTM, sequestome-1; SRSF1, serine/arginine-rich splicing factor 1; SV40, simian virus 40; SVST, SV40 small tumor antigen; TRAIL, tumor necrosis factor-related apoptosis-inducing ligand; TRB3, tribbles homolog 3; TNBC, triple-negative breast cancer; TNFα, tumor necrosis factor-α; TOP2B, DNA topoisomerase IIβ; t-RPS6, total RPS6; TSC, tuberous sclerosis; ULK1, Unc-51-like kinase 1; URM1, ubiquitin-related modifier 1; UTR, untranslated region; UV, ultraviolet; V-ATPase, vacuolar proton-ATPase; VIN, vulvar intraepithelial neoplasia; VHL, von Hippel-Lindau disease tumor suppressor; VSCC, vulva squamous cell carcinoma; WIP1, wild-type p53-induced phosphatase 1; WNT3A, wingless-related integration site family member 3A; YY1, Yin-Yang 1; ZBED1, zinc finger BED domain-containing protein 1.
